# A Dynamic View of Trauma/Hemorrhage-Induced Inflammation in Mice: Principal Drivers and Networks

**DOI:** 10.1371/journal.pone.0019424

**Published:** 2011-05-10

**Authors:** Qi Mi, Gregory Constantine, Cordelia Ziraldo, Alexey Solovyev, Andres Torres, Rajaie Namas, Timothy Bentley, Timothy R. Billiar, Ruben Zamora, Juan Carlos Puyana, Yoram Vodovotz

**Affiliations:** 1 Department of Sports Medicine and Nutrition, University of Pittsburgh, Pittsburgh, Pennsylvania, United States of America; 2 Department of Mathematics, University of Pittsburgh, Pittsburgh, Pennsylvania, United States of America; 3 Department of Surgery, University of Pittsburgh, Pittsburgh, Pennsylvania, United States of America; 4 Office of Naval Research, Code 34, Arlington, Virginia, United States of America; 5 Center for Inflammation and Regenerative Modeling, McGowan Institute for Regenerative Medicine, University of Pittsburgh, Pittsburgh, Pennsylvania, United States of America; Massachusetts General Hospital and Harvard Medical School, United States of America

## Abstract

**Background:**

Complex biological processes such as acute inflammation induced by trauma/hemorrhagic shock/ (T/HS) are dynamic and multi-dimensional. We utilized multiplexing cytokine analysis coupled with data-driven modeling to gain a systems perspective into T/HS.

**Methodology/Principal Findings:**

Mice were subjected to surgical cannulation trauma (ST) ± hemorrhagic shock (HS; 25 mmHg), and followed for 1, 2, 3, or 4 h in each case. Serum was assayed for 20 cytokines and NO_2_^−^/NO_3_^−^. These data were analyzed using four data-driven methods (Hierarchical Clustering Analysis [HCA], multivariate analysis [MA], Principal Component Analysis [PCA], and Dynamic Network Analysis [DyNA]). Using HCA, animals subjected to ST vs. ST + HS could be partially segregated based on inflammatory mediator profiles, despite a large overlap. Based on MA, interleukin [IL]-12p40/p70 (IL-12.total), monokine induced by interferon-γ (CXCL-9) [MIG], and IP-10 were the best discriminators between ST and ST/HS. PCA suggested that the inflammatory mediators found in the three main principal components in animals subjected to ST were IL-6, IL-10, and IL-13, while the three principal components in ST + HS included a large number of cytokines including IL-6, IL-10, keratinocyte-derived cytokine (CXCL-1) [KC], and tumor necrosis factor-α [TNF-α]. DyNA suggested that the circulating mediators produced in response to ST were characterized by a high degree of interconnection/complexity at all time points; the response to ST + HS consisted of different central nodes, and exhibited zero network density over the first 2 h with lesser connectivity vs. ST at all time points. DyNA also helped link the conclusions from MA and PCA, in that central nodes consisting of IP-10 and IL-12 were seen in ST, while MIG and IL-6 were central nodes in ST + HS.

**Conclusions/Significance:**

These studies help elucidate the dynamics of T/HS-induced inflammation, complementing other forms of dynamic mechanistic modeling. These methods should be applicable to the analysis of other complex biological processes.

## Introduction

The advent of multi-dimensional datasets derived from dynamic experiments on complex biological systems has resulted in a deluge of data, but this massive increase in data has not necessarily translated to enhanced mechanistic understanding [1]. One field in which a plethora of both *in vitro* and *in vivo* data has not been directly linked to a dynamic, mechanistic understanding is the field of acute inflammation induced by trauma/hemorrhage and related phenomena such as sepsis [2], [3], [4], [5], [6].

Traumatic injury, often accompanied by hemorrhage, represents the most common cause of death for young people, as well as a significant source of morbidity and mortality for all ages [7]. Hemorrhage and trauma, like infection, are insults that induce an acute inflammatory response involving a coordinated mobilization of numerous cells and molecules, with repercussions on all organ systems [8], [9], [10], [11]. Importantly, an adequately robust, early inflammatory response appears to be crucial for the survival of both trauma patients and experimental animals subjected to T/HS [12]. However, the inflammatory response can also compromise healthy tissue, further exacerbating inflammation [11], [13]. Numerous prior studies have documented both dynamic changes in circulating inflammatory mediators [14], [15], but these studies have generally led to reductionist hypotheses rather than defining networks of interactions.

The complex nature of the response to T/HS, with its many redundant and overlapping pathways and mediators [16] does not lend itself to a simple reductionist analysis, especially when there is limited or no experimental constraints on this system [2], [3], [4]. We hypothesize that these multiple mechanisms of inflammation, operating at different time scales, contribute to the complexity of the post-T/HS inflammatory response. We have gained insights into this complex response using mechanistic mathematical models that recapitulate known mechanisms of acute inflammation in various settings of trauma [17], [18], [19], [20], [21], [22]. The mathematical models described in these earlier studies were based on consensus interactions gleaned from the literature.

Herein, we applied a set of novel, data-driven methods to dynamic, multi-dimensional data derived from a highly-precise, survivable mouse model of T/HS in order to discern novel mechanistic interactions directly from data. These studies demonstrate that survivable trauma elicits an inflammatory response as early as 1 h post-injury. Our results also suggest that the response to low-level trauma is driven by particular cytokines in a complex and well-ordered manner, while the addition of survivable HS leads to the elaboration of distinct inflammatory mediators as part of a much less complex and less organized response.

## Materials and Methods

### Experimental T/HS

This study was approved by the Institutional Animal Care and Use Committee of the University of Pittsburgh (protocol No. 1003645) and was conducted in accordance with the National Institutes of Health Guidelines for the Care and Treatment of Small Laboratory Animals. All studies were initiated only following a two-week acclimatization period at the University of Pittsburgh, Biomedical Science Tower Animal Facility, with access to food and water *ad libitum.* Fifty-four Male C57BL/6 mice (Charles River Laboratories, Raleigh, NC) weighting 25–30 grams underwent surgical preparation under anesthesia with isoflurane and Nembutal (70 mg/K). Animals were either untreated (n = 6) or were cannulated and divided into four groups (n = 6 mice per group), subjected to 1, 2, 3 or 4 h sham procedure (surgical cannulation trauma only; ST) or 1, 2, 3 and 4 h of HS in addition to this surgical cannulation trauma (ST + HS). ST + HS was carried out using a hardware/software platform for computerized, closed-loop HS in mice described previously [21], described in greater detail in Document S1.

### Quantification of serum analytes

A central goal of this study was to assess the dynamics of several key inflammatory analytes, which are representative of the acute inflammatory response and which have been shown to be modulated in humans that have undergone trauma/HS [17], [23]. Accordingly, blood was collected at all experimental time points in order to obtain serum for analysis of circulating inflammatory analytes. Twenty cytokines and chemokines (basic fibroblastic growth factor [bFGF], granulocyte-macrophage colony stimulating factor [GM-CSF], interferon [IFN]-γ, IL-1α, IL-1β, IL-2, IL-4, IL-5, IL-6, IL-10, IL-12p40/p70, IL-13, IL-17, IP-10, KC, monocyte chemotactic protein-1 [MCP-1], MIG, macrophage inflammatory protein-1α (CCL-3) [MIP-1α], TNF-α, and basic VEGF) were assessed using the Luminex™ multiplexing platform (MiraiBio, Alameda, CA) using the BioSource 20-plex™ mouse cytokine bead set (BioSource-Invitrogen, San Diego, CA) as per manufacturer's specifications. The nitric oxide reaction products NO_2_^−^/NO_3_^−^ were assessed using the nitrate reductase kit (Cayman Chemical, San Diego, CA) as per manufacturer's specifications.

### Data analysis and data-driven modeling

**Figure 1:**
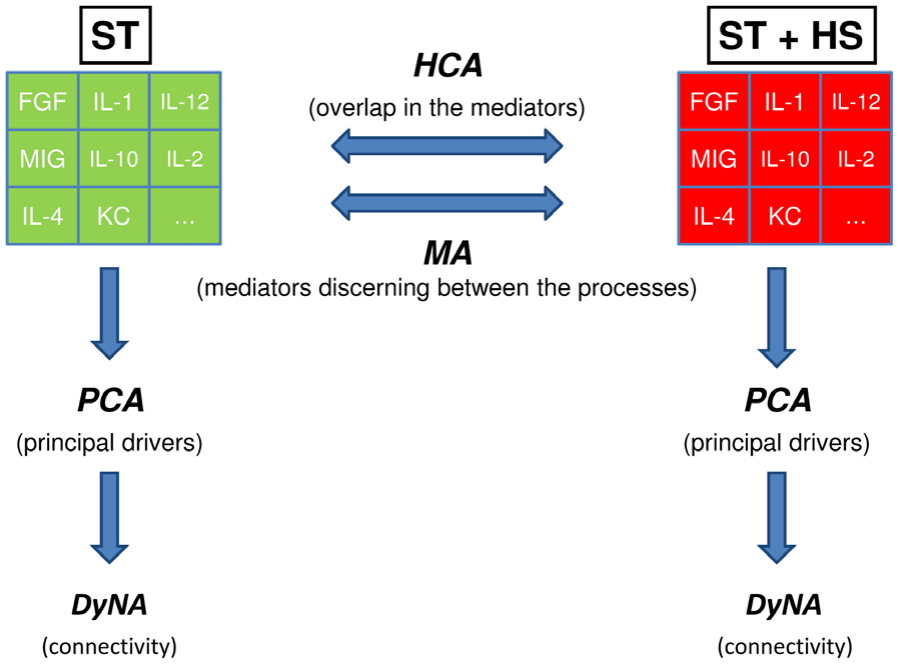
Schematic of analyses utilized in the present study. Mice were subjected to ST ± HS followed by measurement of cytokines, chemokines, and NO_2_^−^/NO_3_^−^ as described in the *Materials and Methods*.

The following analyses were carried out in an attempt to discern differences in, and derive mechanistic insights from, changes in inflammatory mediators across experimental procedures. The null hypothesis for all of these studies was that inflammatory mediators could not segregate ST from ST + HS. The schematic of the analyses and their respective goals is depicted in Fig. 1.

#### Data analysis: Univariate analysis and Analysis of Variance

Univariate analysis explores individual variable in a data set. It describes the pattern of response to the variable. Our response variables are the 21 inflammatory mediators described above, and *Procedure* and *Time* are the controllable input variables on which the mediators are assumed to depend. Differences in individual mediators were examined by performing independent univariate analyses, i.e., the mean and t-difference of these mediators ST vs. ST + HS at each time point (1, 2, 3, and 4 h). The results of this analysis are depicted in Tables S1 – S3 and Fig. S1, and the most significant variables are depicted in Table 1. Table S4 encapsulates the significance of the two factors (*Procedure* and *Time*), as well as their interaction, in each of the five most discriminating mediators. This was achieved by using a linear model to model each of the five responses as a function of *Procedure*, *Time*, as well as their *Interaction*. The mediator IL-12p40/p70 (IL-12.total), is explicitly modeled in a detailed example of this procedure, as described in the *Results* section, and the observed and fitted values are displayed in Fig. S2A. Since the mediators were generally correlated, (see Table S5), we developed a tri-variate linear model for IL-12.total, KC, and MIG as a function of *Procedure* and *Time*. The model fits are displayed in Figs. S2B-D.

**Table 1:** Top five significantly different cytokine in ST vs. ST + HS.

	IL-12.Total	IL-6	IP-10	KC	MIG
**1 h**	2.43	1.76	3.31	2.20	2.07
**2 h**	2.76	2.18	1.35	0.99	3.13
**3 h**	6.88	2.24	2.22	2.96	6.35
**4 h**	3.04	3.08	2.17	4.84	1.46
**Overall**	4.75	2.65	3.00	3.31	4.42

Mice were subjected to ST ± HS followed by measurement of cytokines, chemokines, and NO_2_^−^/NO_3_^−^ as described in the *Materials and Methods*. The data were subjected to univariate analysis as described in the Materials and Methods; the top five inflammatory mediators most significantly different between ST and ST + HS are depicted.

In the sections below, we describe the approach utilized to determine if inflammatory mediators could predict the *Procedure* (ST or ST + HS) to which experimental animals were subjected.

#### Data-driven modeling: Hierarchical clustering analysis [HCA] of cytokine data

The goal of this analysis was to highlight the natural variability, as well as any overlap, in inflammatory mediators from animals subjected to ST or ST + HS. Hierarchical clustering is a simple and unbiased clustering method which aims to build a hierarchy of clusters. The limitation is the cluster must be built pairwise; since it is purely based on the similarity between the data, the cluster may lack biological relevance [24]. This analysis was performed for all the inflammatory analytes in the ST + HS and ST groups; the 6 samples from completely untreated mice were omitted from this analysis. Each row of the data matrix corresponds to a sample from a single mouse, and each column corresponds to an inflammatory analyte (21 total: 20 cytokines/chemokines along with NO_2_^−^/NO_3_^−^). The magnitudes of these values were log-transformed and indicated by colors. The dendrogram (a branching diagram used to show relationships between members of a group) on the y-axis shows the similarities among samples according to their correlation measures (the correlation between the inflammatory mediators profiles) across all analyte values. The calculation is performed by using the Bioinformatics Toolbox in Matlab® 7.6.0.

#### Data-driven modeling: Multivariate analysis ([MA] Assessing the predictive value of each mediator as to defining whether a given animal was subjected to ST or ST + HS)

The goal of this analysis was to determine which inflammatory mediators reach levels sufficiently different following each insult so as to discriminate between ST and ST + HS. To do so, a multivariate statistical model was developed that takes as input the cytokine readings in mice and yields as output the probability that the mouse in question belongs to a specific group: ST + HS or ST only. The model uses an additive, main effects only design. The experimental procedures ST and ST + HS represent a binary response. Specifically, if *p* denotes the probability that a mouse is subjected to ST + HS, we express the log-odds ratio as a multiple regression of the independent variables
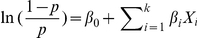



where the β's are unknown parameters subject to estimation, and the X's represent the predictor variables (selected inflammatory mediators).

Several predictive classes of models were investigated, and the logistic family was found to be the best suited for this task. The individual predictive ability of each mediator was ranked by using the corresponding p-values derived through the logistic model fit involving that sole mediator as input variable. In addition, a predictive model involving just two cytokines, *IL-12.total* and *MIG* as predictor variables, was also constructed, as a preferred overall predictive model. In this case, 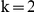 and 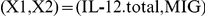 is the vector of the two variables. Maximum likelihood estimation yields estimates for the model coefficients (the β's) and exponentiation of the log-odds function then yields the estimates of the individual probabilities. The model was developed on 80% of the available data and used to predict the remaining 20%.

#### Data-driven modeling: Principal Component Analysis [PCA]

The goal of this analysis was to identify the subsets of mediators (in the form of orthogonal normalized linear combinations of the original mediator variables, called principal components) that are most strongly correlated with a given experimental procedure (ST or ST+HS), and that thereby might be considered principal drivers of each response. PCA is a non-parametric statistical method of reducing a multidimensional dataset to a few principal components [25]. These are the components that account for the most variability in the dataset. The underling hypothesis is that a mediator that changes during a specific process is important to that process. If the mediators that change more than other mediators, then it is are more important. This method allows us to identify the mediators that account for the most change, or variance, in the dataset. The limitation is that some principal components may lack biological relevance [24]. To perform this analysis, the cytokine and NO_2_^−^/NO_3_^−^ data were first normalized for each cytokine (i.e. a given value divided by the maximum value for a given inflammatory mediator), so that all cytokine levels were converted into the same scale (from 0 to 1). In this way, any artifactual effects on variance due to the different ranges of concentration observed for different cytokines were eliminated. Only sufficient components to capture at least either 70% or 95% of the variance in the data were considered. From these leading principal components, the coefficient (weight) associated with each cytokine was multiplied by the eigenvalue associated with that principal component. This product represented the contribution of a given cytokine to the variance accounted for in that principal component. The overall score given to each cytokine is the sum of its scores in each component. This gives a measure of a cytokine's contribution to the overall variance of the system. The cytokines with the largest scores are the ones who contributed most to the variance of the process being studied. More specifically, the overall PCA score was calculated in the following way: 
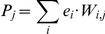
, where i is the index of component and j is the index of cytokine. W_i,j_ is the amount that how much j^th^ cytokine contributes to the i^th^ component. e_i_ is the percentage of total variance accounted by i^th^ component. The Matlab® code for this analysis is included in Document S2.

**Data-driven modeling: Dynamic Network Analysis [DyNA].** The goal of this analysis was to gain insights into dynamic changes in network connectivity of the inflammatory response to ST and ST + HS over time. The mathematical formation of this method is essentially to calculate of the correlation among the variables by which we can examine their dependence. To do so, cytokine networks were created in adjacent 1–h time periods (0–1 h, 1–2 h, 2–3 h, and 3–4 h) using Matlab® and Inkscape® software (http://inkscape.org/). In order to be included in the DyNA, a given mediator had to be statistically significantly different from its baseline value (no treatment [time  = 0]; p<0.05 by Student's t-test). Connections in the network were created if the correlation between two nodes (inflammatory mediators) were greater or equal to a threshold of 0.7 (based on a total of 12 samples with 10 degrees of freedom, p<0.05). In the network density calculation, in order to account for network sizes (number of significantly altered nodes) in the adjacent 1–h time periods detailed above, we utilized the following formula: (a minor revision of the one reported by Assenov *et al* [26]). The Matlab® and Inkscape® code for this analysis are included in Document S3.
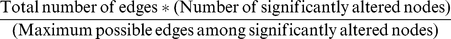



## Results

We initially examined the levels of inflammatory analytes in the serum of C57Bl/6 that were subjected to ST ± HS, to confirm prior studies that have demonstrated elevations in circulating inflammatory analytes (e.g. TNF-α, IL-6, IL-10, NO_2_^−^/NO_3_^−^) following T/HS in mice [17], [21], [23]. Mice were subjected either to surgical cannulation (ST) alone or in combination with bleeding to a target MAP of 25 mmHg (ST + HS) and maintained in that state for 0 (baseline control), 1, 2, 3, or 4 h in each case. Since we attempted to obtain as global a view as possible of the post-T/HS inflammatory response, serum samples were collected at the end of every time point and each sample was assessed for basic FGF, GM-CSF, IFN-γ, IL-1α, IL-1β, IL-2, IL-4, IL-5, IL-6, IL-10, IL-12p40/p70, IL-13, IL-17, IP-10, KC, MCP-1, MIG, MIP-1α, TNF-α, and basic VEGF using a mouse-specific bead set (Table S1). In addition, the NO reaction products NO_2_^−^/NO_3_^−^ (Table S2) were assessed. In total, 21 inflammatory mediators were thus assessed over time in the following experimental scenarios: no treatment (n  = 6 mice per group); 1, 2, 3, or 4 h following surgical cannulation trauma (ST; n  = 6 mice per group); or 1, 2, 3, or 4 h of ST + HS (bleeding to 25 mmHg; n  = 6 mice per group). The raw values of the all of the tested inflammatory mediators are shown in Fig. S1.

**Figure 2:**
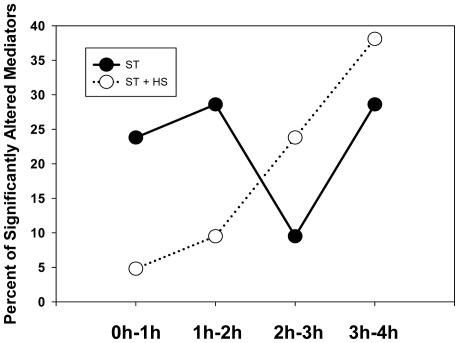
Percent of inflammatory analytes modulated as a function of time and procedure. Mice were subjected to ST ± HS followed by measurement of cytokines, chemokines, and NO_2_^−^/NO_3_^−^ as described in the *Materials and Methods*. In each adjacent 1–h time period (0–1 h, 1–2 h, 2–3 h, and 3–4 h), the statistically significantly altered inflammatory analytes (p<0.05 by Student's t-test) were selected out of the total 21 mediators by comparing the level of a given mediator with its baseline value (no treatment [time  = 0]).

Despite these dynamic changes in inflammation biomarkers as a function of time, we sought to determine if a significant proportion of these 21 mediators were altered as a function of time. This question is especially important for any conclusions that might be drawn regarding principal drivers or dominant networks. Figure 2 shows that nearly 30% (ST) and up to 40% (ST + HS) of all queried mediators were altered over the time course studied. Interestingly, this analysis suggested that ST results in a near maximal alteration of inflammatory mediators between 0–1 h, peaking between 1–2 h, and then dropping between 2–3 h before returning to peak levels between 3–4 h. In contrast, ST + HS resulted in a near-linear increase in inflammatory mediators between 1–4 h, reaching a maximum of ∼40% by 3–4 h.

**Figure 3:**
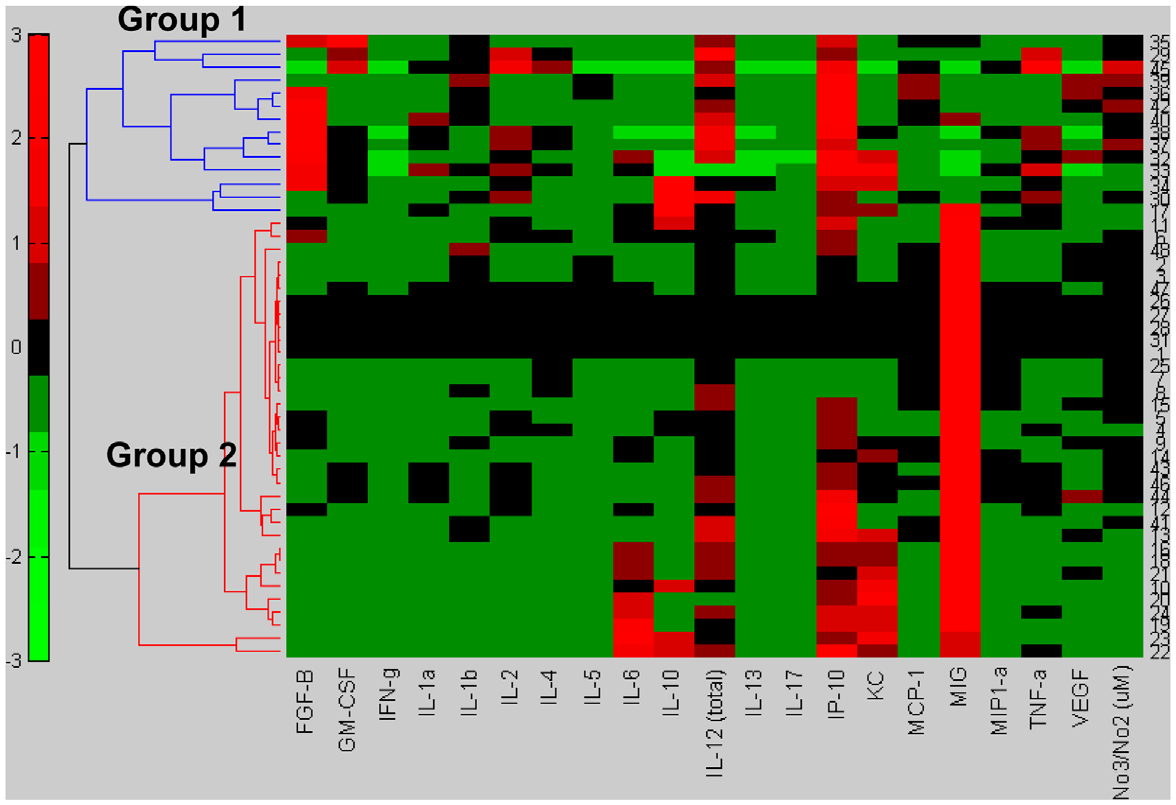
Hierarchical Clustering Analysis of circulating inflammation biomarkers in ST ± HS. Mice were subjected to ST ± HS followed by measurement of cytokines, chemokines, and NO_2_^−^/NO_3_^−^ as described in the *Materials and Methods*. Group 1, depicted in blue, 93% of Group 1 samples are ST and 7% are ST + HS; in Group 2, 32% samples are ST and 68% are ST + HS. A Chi-square test with P<0.001 suggests that the distribution of ST vs. ST+HS animals between Groups 1 and 2 is not random.

**Figure 4:**
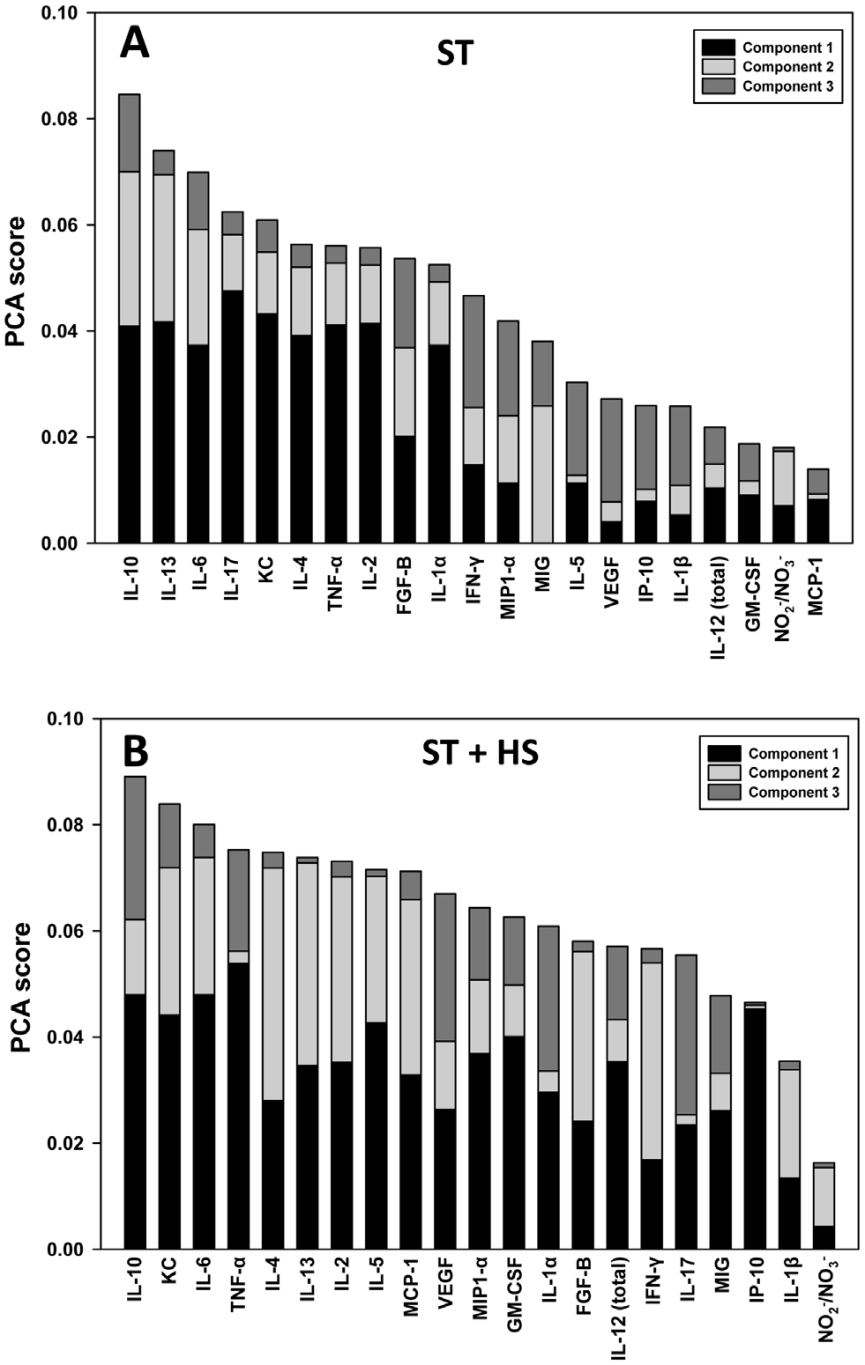
Principal Component Analysis of circulating inflammatory mediators induced by ST ± HS. Mice were subjected to ST ± HS followed by measurement of cytokines, chemokines, and NO_2_^−^/NO_3_^−^ as described in the *Materials and Methods*. The figure shows the sorted overall PCA score for each inflammatory mediator.

**Figure 5:**
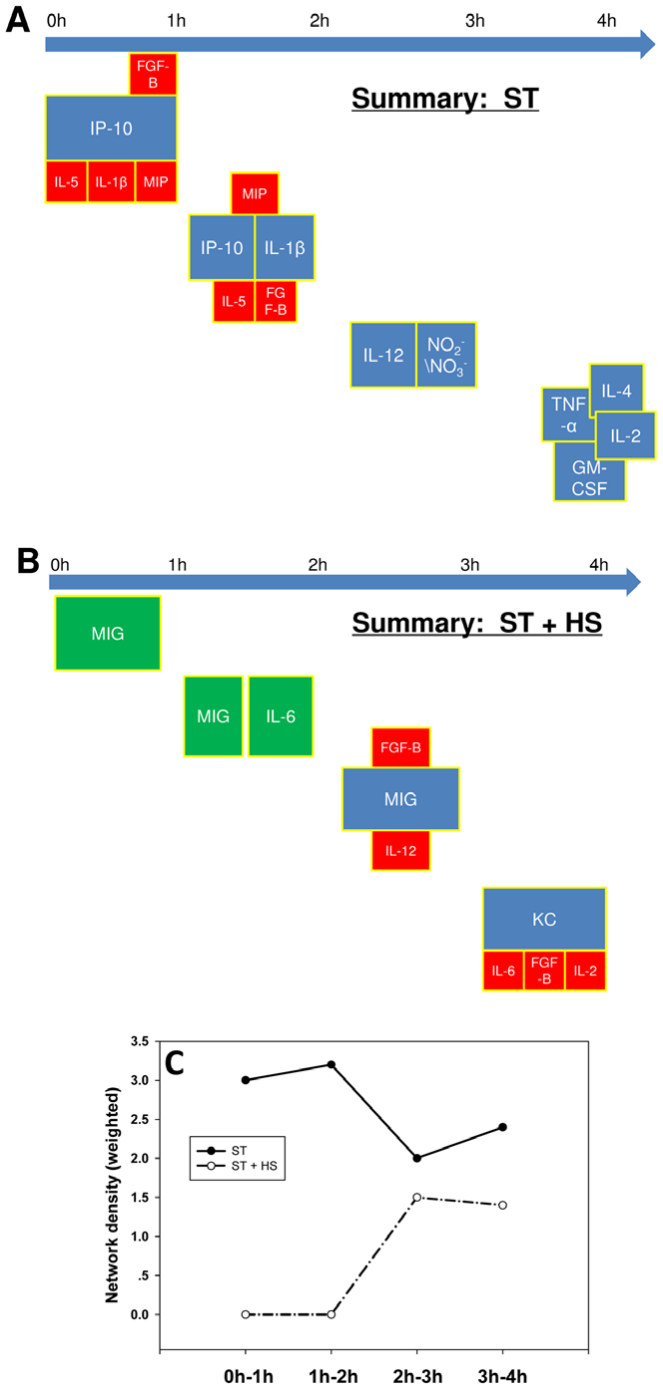
Dynamic Network Analysis summary for ST ± HS. Mice were subjected to ST ± HS followed by measurement of cytokines, chemokines, and NO_2_^−^/NO_3_^−^ as described in the *Materials and Methods*. DyNA was carried out using these data as described in the *Materials and Methods* *. Panel A:* DyNA for ST, during each of the following four time frames: 0–1 h, 1–2 h, 2–3 h, and 3–4 h. *Panel B:* DyNA for ST + HS. In both *Panels A* and *B*, the most connected inflammatory mediators (nodes) are depicted in blue, and the inflammatory mediators linked directly to each central node are depicted in red. The mediators depicted in green are statistically significantly different from their own baseline values (p<0.05), but not correlated with any other mediators. *Panel C:* Network density plot for ST and ST + HS during each of the four time frames (0–1 h, 1–2 h, 2–3 h, and 3–4 h).

To gain a systems perspective on these complex, time-dependent responses to ST ± HS, we carried out univariate analysis, multivariate analysis (MA), hierarchical clustering analysis (Fig. 3), Principal Component Analysis (PCA; Fig. 4), and Dynamic Network Analysis (DyNA; Fig. 5). Initially, we wished to assess the degree of inter-animal and inter-procedure variability between the ST and ST + HS experimental groups. Hierarchical clustering was performed for all the inflammatory analytes in the ST + HS (Fig. 3, samples 1–24) as well as ST (Fig. 3, samples 25–48) groups; the 6 samples from control, untreated mice were omitted from this analysis. Each row of the data matrix corresponds to a sample from a single mouse, and each column corresponds to an inflammatory analyte (21 total: 20 cytokines/chemokines along with NO_2_^−^/NO_3_^−^). The log-transformed magnitudes of these values are indicated by the colors as shown in the color bar (Fig. 3). The dendrogram on the y-axis shows the similarities among samples. .In agreement with prior studies from our group [19], this analysis suggested that circulating inflammatory mediators could to some degree segregate ST from ST + HS. However, a fair amount of overlap was observed in the inflammatory response to ST alone vs. ST + HS: 93% of Group 1 samples were derived from animals subjected to ST, while 7% were derived from animals subjected to ST + HS. In Group 2, 32% samples were derived from animals subjected to ST and 68% were derived from animals subjected to ST + HS. A Chi-square test with P<0.001 suggested that the distribution of ST vs. ST+HS animals between Groups 1 and 2 was not random.

Despite this overlap, we hypothesized that data-driven analyses would uncover distinct features of inflammation in ST vs. ST + HS. We initially employed both univariate and multivariate analyses.

### Univariate analysis of T/HS in mice

We first focused on the time-dependent differences in individual mediators by performing independent univariate analyses. The means of the 21 inflammatory mediators induced in response to ST ± HS in the present study are depicted in Fig. S1 and Tables S1 and S2. Table S3 shows the t-differences of ST + HS - ST at 1, 2, 3, and 4 h, respectively, followed by the t-difference across the entire dataset. In the last row of Table 1, all t-values are significant at the 0.01 level, indicating that all these cytokines distinguish between ST + HS vs. ST when averaging across time. Univariate analysis suggested that Total IL-12 (i.e. the combination of IL-12 p40/p75) best discriminates ST + HS from ST at 3 h, with a t-value of 6.88, and a p-value <0.0001. In this Table, only three entries are not significant at a level of 5%: those less than 1.82 (the quantile associated with the 5% level of significance). Among the five cytokines and chemokines depicted in Table 1, IP-10 is the only one that captures the difference between ST + HS and ST early (at 1 h). As Table 1 indicates, the difference between ST + HS and ST is mostly captured at 3 or 4 h by the other significant variables. Additionally, at the p-value level of 0.01, significant differences occur in Total IL-12, IL-6, IP-10, KC, and MIG. In addition, significant differences at the 0.03 level are also seen in IL-10, TNF-α, and VEGF.

We next carried out an ANOVA for the five responses deemed most significant from the initial univariate analysis (IL-12, IL-6, IP-10, KC, and MIG); the results of this analysis are summarized in Table S4. From the table, we see that the *Procedure* (ST vs. ST + HS) is highly significant. A *Time* effect exists and is significant at a level of 2% for Total IL-12, IL-6, and KC. For these three cytokines, the interaction between *Procedure* and *Time* (*Int*) is found to be significant at level of 3% for Total IL-12, and at a much lower level for IL-6 and KC, as Table S4 indicates. The data indicate that the chemokines KC and MIG are only significantly affected by the *Procedure*, but not by *Time* or *Interaction*.

A more refined model clarifies exactly which interactions between *Procedure* and *Time* account for the IL-12.total response. We fit linear and quadratic time trends, and the following statistical model was generated:
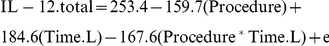



with e denoting a Gaussian random variable with 0 mean, and Time.L, signifying the linear effect of time. All three effects are statistically significant at a level of 0.007. The quadratic time effect Time.Q and the interaction Procedure*Time.Q are not statistically significant (the p-values are 0.31 and 0.28, respectively). This means that IL-12.total grows linearly only (not quadratically) with time, and that this linear time growth depends on the Procedure (explaining the existing interaction). A comparison of the actual Total IL-12 data to the fitted value produced by this model is found in the Fig. S2A.

### Multivariate analysis of T/HS in mice

The five cytokine responses of interest are correlated across all data. The correlation matrix appears in Table S5. Since IL-6 and KC have a correlation exceeding 90%, and IP-10 is the cytokine of least relevance among the five, we shall undertake the construction of a (two-way) Multivariate Analysis of Variance (MANOVA) model by using cytokines (IL-12.total, KC, MIG) as a trivariate response. The explanatory factors are, as in the univariate studies, *Procedure* and *Time*. The model uses all possible interactions between the two factors (it is not assumed additive). The Wilk's lambda is highly significant (p-value <0.0001), allowing us to reject the hypothesis that the means are equal across the levels of the two factors. The trinomial response and trivariate fits of the above model are displayed in Fig. S2.

### Using inflammatory mediators to discern the procedure to which experimental animals were subjected

A major goal of this analysis is to determine a model that uses mediators as predictors for the experimental *Procedure* (ST or ST + HS) to which mice were subjected. We first assessed the importance of each mediator in predicting the *Procedure*, by fitting a logistic predictive model using that sole mediator as predictor. Table S6 summarizes this analysis based on the fitting of predictive logistic models for each mediator. This table shows a partition of the 14 cytokines into three groups, based upon their individual predictive ability (ranked by the p-values in Table S6). The first group, consisting of IL-12.total, MIG, and IP-10 comprises the best predictors; the second-best group consists of KC and VEGF. The other cytokines listed have less dramatic, but still statistically significant, effects at the 10% level. It is interesting to note that GM-CSF has a p-value of 1.4% and varies in opposite direction from the other 12 significant cytokines when comparing ST +HS to ST.

With the relevant mediators for the *Procedure* identified, we embarked on the task of generating a simple predictive model that identifies accurately predicts the *Procedure* as a function of the relevant mediators. The specific logistic predictive model that results is:
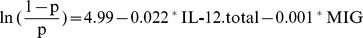



with the estimated coefficients carrying p-values of 0.0005, 0.0019, 0.0082, respectively.

As depicted in Table S7, the model correctly distinguished ST + HS from ST in 46 out of 48 cases, a success ratio of 96%. This is highly significant when compared to a random assignment based on a hypergeometric distribution, which would yield, on average, only 24 out of 48 mice correctly classified. Under the hypothesis of random assignment of each mouse to ST + HS or ST, the chance of obtaining 46 or more correct assignments out of 48 is less than 0.001%. This model is, therefore, a helpful tool in distinguishing successfully between ST + HS and ST from the readings of IL-12.total and MIG.

### Principal component analysis (PCA)

We next attempted to leverage the insights gained from statistical analyses into mechanistic insights regarding the dynamics of inflammation following T/HS. We initially utilized PCA in order to identify the subsets of mediators that are most strongly correlated with ST or ST + HS, and that thereby might be considered principal drivers of each response. Importantly, PCA is based on time-dependent changes in variance, and therefore we hypothesized that this analysis would yield insights into the dynamic responses of the various inflammatory mediators. Figure 4 shows the top three principal components for ST (Fig. 4A) and ST + HS (Fig. 4B). Fig. S3 shows two other variants of this analysis, namely the principal components that comprise 70% (Figs. S3A and S3C) and 95% (Figs. S3B and S3D) of the total variance of the total variance, respectively. This analysis suggested that the principal cytokines driving the response to ST were IL-10, IL-13, and IL-6 (Figs. 4A, S3A, and S3C). In contrast, the principal cytokines characterizing ST + HS were IL-10, KC, and IL-6 (Figs. 4B, S3B, and S3D).

### Dynamic Network analysis (DyNA)

Finally, we wished to expand our mechanistic analysis further by examining the time-dependent evolution of cytokine networks inferred from correlated changes in circulating inflammatory mediators; we refer to this process as Dynamic Network Analysis (DyNA). We wished not only to determine which networks were present at various time intervals, but also to assess the total degree of connectivity at each of these intervals. Fig. S4 shows the detailed DyNA results for ST and ST + HS in the different time periods; this analysis is summarized in Figs. 5A and 5B. DyNA suggested that the central nodes were shifting rapidly post-ST, from IP-10 (0–1 h), to IP-10/IL-1β(1–2 h), then IL-12/NO_2_^−^/NO_3_^−^ (2–3 h), and lastly TNF-α/ IL-4/IL-2/GM-CSF (3–4 h) (Figs. 5A and S4). In contrast, the central nodes over the same time ranges in ST + HS were MIG (0–1 h), MIG/IL-6 (1–2 h), MIG (2–3 h), and lastly KC (3–4 h) (Figs. 5B and S4).

Finally, we wished to go beyond an examination of inflammatory mediators and assess the global state of inflammatory networks, by quantifying the degree of network connectivity as a function of time following ST ± HS (Fig. 5C). The ST response was characterized by a high network density at all time points. In stark contrast, ST + HS network density was zero over the first 2 h and, though network connectivity increased thereafter, it remained lower than that of ST at all time points (Fig. 5C).

## Discussion

Detailed cellular and molecular analyses explored in isolation have provided valuable insights into the pathobiology of sepsis and T/HS, but have often been limited in their global applicability [3], [4]; this is a problem shared with many complex, dynamic biological systems [1]. Data-driven analyses of genomic [27], [28], [29] and proteomic [30] studies, along with mechanistic computational modeling based on measurements of circulating inflammatory mediators [17], [18], [19], [21], have yielded insights into the pathophysiology of T/HS. Herein, have sought to link two classes of studies, namely data-driven, pattern-oriented analyses of high-content datasets [27], [28], [29], [30] and mechanism-based computational modeling [17], [18], [19], [21], in order to gain quantitative, mechanistic insights into the complexity of acute inflammation [31], [32], [33]. We suggest that the approaches outlined herein have broad applicability in biological studies, both *in vitro* and *in vivo*.

In the studies described herein, mice were subjected to highly precise and reproducible experimental T/HS (bleeding down to 25 mmHg without resuscitation) for 1–4 h using a computerized hemorrhage system described previously [21], to the sham cannulation procedure (surgical cannulation trauma, ST) for the same periods of time, or in completely non-manipulated animals. The 1–4 h time range was chosen since mice can survive this particular combined ST + HS insult for this length of time, and so the present study essentially surveys the insult severity range over which the host's responses should remain robust. Twenty-one inflammatory mediators representative of various manifestations of acute inflammation were assessed over this time course in these mice. Depending on the time point assessed, up to 40% of these circulating inflammation biomarkers were altered relative to the levels found in resting animals (Fig. 2), suggesting that the analytes chosen were relevant to the experimental paradigm of T/HS utilized in these studies.

We hypothesized that the data regarding the dynamic evolution of these 21 mediators/biomarkers could be analyzed using data-driven modeling approaches, following the framework depicted in Fig. 1, in order to yield mechanistic insights regarding the roles of these mediators in T/HS. The methods we utilized can be separated into two broad categories: analyses that attempt to discern differences across the experimental procedures (ST vs. ST + HS), and analyses that attempt to define mechanistic drivers within ST or ST + HS.

In the first category (across experimental procedures), we employed two distinct methods. Hierarchical Clustering Analysis was used to examine both the natural variability of and the overlap in circulating inflammatory mediators in animals subjected to ST or ST + HS. This analysis highlighted the relatively high degree of overlap between ST and ST + HS. A prior study from our group had also described this large overlap in the pathways induced by ST and ST + HS, though this prior study only examined TNF-α, IL-6, IL-10, and NO_2_^−^/NO_3_^−^ as well as changes in the liver transcriptome [19]. The other analyses across experimental procedures were multivariate and univariate analyses. These approaches were utilized in order to test the hypothesis that defined inflammatory mediators reach levels sufficiently different following ST vs. ST+HS so as to discern between each insult.

Recent studies have reported on the use of multiplexed cytokine analysis coupled with multivariate regression modeling in mouse models of inflammation, e.g. colitis [34], [35]. In our study, we focused on circulating inflammatory mediators rather than examining cellular or tissue responses, since the inflammatory response to T/HS can spill out into the systemic circulation and, when dysregulated, is associated with MODS and death [14], [15], [36], [37], [38], [39], [40]. Of the various circulating mediators that can be detected systemically following T/HS, we concentrated on cytokines, chemokines, and NO reaction products. Cytokines are a broad class of protein hormones that mediate inflammatory and immune responses in a complex, context-sensitive manner [11], [41]. Major cytokines that participate in the response to trauma include TNF-α, IL-1β, IL-2, IL-6, IL-8 [36], [42], [43], IL-4 [44], and IL-18 [45]. The nominally anti-inflammatory cytokine IL-10 counteracts the effects of the nominally pro-inflammatory cytokines IL-1β, IL-6 and TNF-α in the setting of T/HS [46]. Chemokines are produced by macrophages, lymphocytes, neutrophils and dendritic cells and mediate various functions of these cells, including recruitment of other cells [47], [48]. Recent studies suggest that chemokines play important roles following T/HS [49], [50], [51]. The free radical NO, when produced at high levels by the inducible NO synthase and typically detected in biofluids as its reaction products NO_2_^−^/NO_3_^−^, is a critical mediator of post-T/HS inflammation [23]. Accordingly, we examined these mediators as well as others (for example, non-classical cytokines/growth factors such as VEGF [52], [53] that have been implicated in sepsis-associated acute inflammation and MODS) in an attempt to assess post-T/HS as broadly as possible in an experimental setting. To reduce experimental variability as much as possible, we utilized a highly reproducible, computerized platform for automated HS in mice that we have used recently in conjunction with mechanistic mathematical modeling of post-T/HS inflammation [21]. Notably, multiple clinical studies have utilized univariate and multivariate analyses to suggest that levels of several of these inflammatory mediators, such as IL-6, IL-8 and IL-10 (along with mediators not measured here, such as soluble TNF-α receptors and damage-associated molecular pattern [DAMP] molecules such as HMGB1), correlate closely with severity of injury and complication rates [54], [55], [56], [57], [58], [59], [60], [61], [62], [63].

In the second category (within a given experimental procedure), PCA was employed in order to discern the main drivers of inflammation and DyNA was utilized in order to define the principal (most connected) nodes being elaborated dynamically as a function of pro-inflammatory insult. The hypothesis underlying the use of PCA was that such main drivers might act “behind the scenes”, and be discerned as those mediators exhibiting the greatest, insult-specific, time-dependent variance. Thus, these principal mediators are hypothesized to define a given experimental procedure across the entire time range studied. It is therefore entirely possible that principal mediators defined thus may not reach statistical significance, since they may carry out their function for a limited period of time and drive the production of other mediators that would in fact remain statistically elevated to a degree sufficient to be detected by MA. Though utilized in a manner somewhat similar to PCA, DyNA was used to gain insights into dynamic changes in network connectivity of the inflammatory response to ST and ST + HS over time, allowing for insights that are difficult, if not impossible, to gain from any of the other data-driven analyses utilized in this study.

We gained several insights from our network analysis. For example, the earliest pro-inflammatory mediators in our mechanistic mathematical models of post-T/HS inflammation is TNF-α, with IL-6 elaborated fairly soon afterwards [17], [18], [19], [21]. Based on DyNA, we find that TNF-α does not appear as an important node until 3–4 h post-ST, while IL-6 is elevated early post-ST + HS. Rather, chemokines such as IP-10 and KC appear to drive the inflammatory response at an earlier stage. This hypothesis is supported by the known central role of chemokines in acute inflammation [47], [48], including T/HS [49], [50], [51]. For example, KC appears to be a central node in the response to ST + HS; Frink *et al* have shown that post-T/HS inflammation and organ damage can be ameliorated by neutralization of KC [50]. The discriminatory power of KC and IL-12 to distinguish ST from ST + HS may point to a role of neutrophils [64] and Th17 cells [65] in trauma, in agreement with prior literature [66], [67].

Beyond such mediator-focused insights, the DyNA studies also uncovered an additional dimension of information about the connectivity of the early inflammatory response to T/HS, namely that the response to a minor trauma (ST) appeared well-ordered and was driven by defined networks orchestrated by chemokines and cytokines. In contrast, the response to that same minor trauma in the presence of HS (ST + HS) was characterized by a complete lack of connectivity among mediators in the first 2 h. Though the degree of connectivity appeared to recover, the networks involved in this attempt at recovery were distinct from those present in the mice not subjected to combined T/HS. Intriguingly, a comparison of network density / complexity over time suggested a “mirror image” pattern when comparing ST vs. ST + HS. While we do not wish to over-interpret this aspect of our data, such a pattern may imply that baseline inflammatory connectivity is initially perturbed upwards (more complexity) by ST, while the addition of HS perturbs baseline connectivity downward (lower complexity) to approximately the same degree. Over time, both responses appear to return towards baseline connectivity, with inflammatory connectivity in ST still remaining higher than ST + HS. We hypothesize that this difference is due to the presence of HS and not to the animals' being near death, since our prior experience [17], [18], [19], [21], [23], [68], [69], [70] suggests that mice are able to tolerate this duration of HS at 25 mmHg. We have recently demonstrated that multiple physiological networks, inferred by data-driven algorithms by examining the liver transcriptome post-T/HS, are differentially modulated by ST and ST + HS (along with subsequent resuscitation; Edmonds *et al*, submitted).

Each of the analyses we performed served a distinct purpose, and therefore these analyses were expected to provide complementary, rather than identical, results. We also expected to find some concordance with our prior mechanistic mathematical modeling of T/HS in mice. Importantly, using the above-described methods, the difference between ST and ST + HS in this experimental model could clearly be distinguished over time, based on certain inflammatory mediators (as well as the mediators that correlated highly with these distinguishing mediators, namely IL12-total, MIG, KC, IL6, and IP10. In addition, the finding by MA that total IL-12 was a good discriminator of ST vs. ST + HS at 3 h is in accord with the DyNA results, which suggests an interaction of IL-12 with NO_2_^−^/NO_3_^−^ between 2 and 3 h post-ST. Interestingly, Diefenbach *et al* have previously described a crucial role for iNOS-derived NO for IL-12 signaling [71]. On another level, our results suggest that the particular type and connectivity of a given individual response to T/HS may predispose that individual to one of a series of outcomes (e.g. life and death). We have recently shown that swine the elaborate an adequately robust TNF-α response to experimental ST + HS (in fact, to ST alone) survive following post-HS resuscitation, while animals that have little or no TNF-α response do not [12]. The present studies may extend this observation to networks of inflammatory mediators and to the degree of connectivity of these networks.

We suggest that data at the mRNA and protein levels, combined with data-driven methods such as those described in this study, may facilitate further mechanistic modeling of the dynamics of acute inflammation as well as driving clinically-relevant advances [31], [32], [33]. For example, we have carried out PCA on Luminex™ measurements of cytokines in the cerebrospinal fluid of traumatic brain injury patients, and constructed mechanistic, equation-based computational models based on the presumed principal drivers [32] (Solovyev *et al*, unpublished observations). Even in the absence of further mechanistic modeling, techniques such as PCA can yield potentially useful diagnostic information in the setting of T/HS. We have shown that PCA carried out in a patient-specific manner based on data obtained in the first 24 h post-T/HS can be used in combination with HCA to define patient sub-groups that differ in organ damage, whereas the raw cytokine were insufficient for such patient segregation [33](Ghuma et al, unpublished). In this study, a large degree of inflammatory and outcome variability could be observed using HCA in a cohort of 25 T/HS patients who were all survivors, leading to an inability to define naturally-occurring groups. Yet, defined patterns in the early (within 24 h post-injury) time course of inflammation biomarkers could be identified via PCA (those cytokines that contribute to 95% of the variance in the patient-specific time course data). These patterns segregated the 25 patients into defined sub-groups exhibiting distinct levels of organ dysfunction; moreover, these patient sub-groups, defined within the first 24 h post-injury, persisted with distinct levels of organ damage for several days [33] (Ghuma *et al*, manuscript in preparation).

Several limitations are associated with our study. One central limitation may revolve around the possible confounding role of anesthesia in our analyses. Prior analyses have suggested that anesthesia may affect inflammatory and related physiological responses [72], [73], [74], [75], [76], [77], [78].

Thus, at least some of the inflammatory response associated with either ST or ST + HS may be due to (or modulated by) the anesthesia used for both procedures. Another limitation concerns the lack of certain key mediators and biomarkers in T/HS, e.g. DAMP's such as HMGB1 or soluble TNF-α receptors. Another limitation of the interpretation of our study is that insult-specific mediators defined by MA may reach statistically different levels not because they are necessarily primary drivers of ST or ST + HS, but perhaps because they are induced to the greatest degree or for the longest duration. Despite these limitations, we suggest that mechanism-focused data-driven analyses based on time-varying, high-content datasets will serve to generate hypotheses regarding the induction and propagation of inflammation, and eventually yield insights into novel therapies.

## Supporting Information

Figure S1 **Inflammatory mediators induced by ST** ± **HS.** Mice were subjected to ST ± HS followed by measurement of cytokines, chemokines, and NO_2_^−^/NO_3_^−^ as described in the *Materials and Methods*. Data are shown as mean ± SEM. Asterisks indicate P<0.05 compared with baseline. Crosses indicate P<0.05 compared with ST
(PPT)

Figure S2 **Observed and fitted values for IL-12, KC and MIG.** *Panel A*: ANOVA (univariate model) fit for IL-12. *Panel B*: MNOVA (trivariate model) fit for IL-12. *Panel C*: MNOVA (trivariate model) fit for KC. *Panel D*: MNOVA (trivariate model) fit for MIG.
(PPT)

Figure S3 **Additional principal component analyses of ST** ± **HS.** The PCA described in Fig. 3 was repeated, with the number of principal components adjusted to account for 70% (*Panels A* and *C*) or 95% (*Panels B* and *D*) of the total variance.
(PPT)

Figure S4 **Dynamic network analysis of circulating inflammatory mediators following ST** ± **HS.** Mice were subjected to ST ± HS followed by measurement of cytokines, chemokines, and NO_2_^−^/NO_3_^−^, followed by Dynamic Network Analysis as described in the *Materials and Methods*. Red nodes indicate that the mediator is statistically significantly different from its baseline value (no treatment [time  = 0]; p<0.05). White nodes indicate no significant change compared to no treatment (time  = 0). Green edges signify a positive correlation and blue edges signify a negative correlation. *Panel A*: Dynamic networks between 0–1 h. *Panel B*: Dynamic networks between 1–2 h. *Panel C*: Dynamic networks between 2–3 h. *Panel D*: Dynamic networks between 3–4 h.
(PPT)

Table S1 **Circulating cytokines and chemokines from mice subjected to ST** ± **HS.** Mice were untreated, subjected to ST for the indicated times, or subjected to ST + HS for the indicated times. Serum was obtained following euthanasia and assayed for the indicated cytokines and chemokines using Luminex™ as described in the *Materials and Methods*. Values are in pg/ml and are given as mean ± SEM. *compared with baseline, P<0.05. †compared with ST, P<0.05. Levene statistic is calculated for variance test and it suggests that the equal variance assumption is rejected with P<0.05. Then one-way ANOVA post Hoc is performed by using Games-Howell test for unequal variances. There are total 9 groups with n = 6 in each group.
(DOC)

Table S2 **Circulating NO_2_^−^/NO_3_^−^ values from mice subjected to ST** ± **HS.** Mice were untreated, subjected to ST for the indicated times, or subjected to ST + HS for the indicated times. Serum was obtained following euthanasia and assayed for NO_2_^−^/NO_3_^−^ using the nitrate reductase method as described in the *Materials and Methods*.
(DOC)

Table S3 **Univariate analysis of circulating inflammatory mediators following ST and ST + HS.** Mice were subjected to ST ± HS followed by measurement of cytokines, chemokines, and NO_2_^−^/NO_3_^−^ as described in the *Materials and Methods*. t-values were calculated for the individual inflammatory mediators at fixed time points (1, 2, 3, and 4 h) and across the entire time range as whole. Since the number of experimental animals for each experimental procedure at each time point is 6, there are 6+6−2 = 10 degrees of freedom for all differences for a fixed time comparison. The last row gives t-values for (mean ST + HS – mean ST) across all time levels; these comparisons carry 24+24 – 2 = 46 degrees of freedom.
(DOC)

Table S4 **Summary of ANOVA models for five significant mediators.** Mice were subjected to ST ± HS followed by measurement of cytokines, chemokines, and NO_2_^−^/NO_3_^−^ as described in the *Materials and Methods*. ANOVA was carried out to determine any interaction effect between Experimental Procedure and Time for all inflammatory mediators studied.
(DOC)

Table S5 **Correlation matrix of the 5 most important mediators.**
(DOC)

Table S6 **Partition of inflammatory mediators by p-values.**
(DOC)

Table S7 **The probabilities of correct identification of the ST procedure, based on the logistic model.** Mice were subjected to ST ± HS followed by measurement of cytokines, chemokines, and NO_2_^−^/NO_3_^−^ as described in the *Materials and Methods*. Logistic model was created based on the levels of these inflammatory mediators. The table depicts the prediction by this model of the Experimental Procedure. Starred entries show misclassification.
(DOC)

Document S1 **Detailed experimental description.**
(DOC)

Document S2 **PCA code.** 1. PCA_instruction.doc: Instruction file for how to conduct PCA. 2. pcaGeneral.m: Main Matlab code for performing PCA. 3. Xticklabel_rotate.m: Matlab code for rotating the labels in the figure.
(RAR)

Document S3 **DyNA code.** 1. DyNA_instruction.doc: Instruction file for how to conduct DyNA. 2. do_all.m: Main Matlab code for producing all network results. It generates labels.csv, sham01.csv, sham12.csv, sham23.csv, sham34.csv, shock01.csv, shock12.csv, shock23.csv and shock34.csv which are used for creating the networks in Inkscape. 3. save.m: Matlab code for saving the correlation result into a file. 4. sham01.m, sham12.m, sham23.m and sham34.m: Matlab code for creation network nodes and correlation matrix for ST in 0–1 h, 1–2 h, 2–3 h and 3–4 h respectively. 5. shock01.m, shock12.m, shock23.m and shock34.m: Matlab code for creation network nodes and correlation matrix for ST + HS in 0–1 h, 1–2 h, 2–3 h and 3–4 h respectively. 6. LoadConnectors,inx, LoadConnector.py, LoadLabels.inx, LoadNodes.py and Networks.py are the Inkscape files for creation Network graphs.
(RAR)
