# Supplementary material for: A Dynamic View of Trauma/Hemorrhage-Induced Inflammation in Mice: Principal Drivers and Networks
Source: PLoS One. 2011 May 10;6(5):e19424. doi: 10.1371/journal.pone.0019424 (PMC3091861; doi:10.1371/journal.pone.0019424)
Supplement: Figure S1 — Inflammatory mediators induced by ST ± HS. Mice were subjected to ST ± HS followed by measurement of cytokines, chemokines, and NO2 −/NO3 − as described in the Materials and Methods . Data are shown as mean ± SEM. Asterisks indicate P<0.05 compared with baseline. Crosses indicate P<0.05 compared with ST (PPT) [file pone.0019424.s001.ppt]

## Slide 1
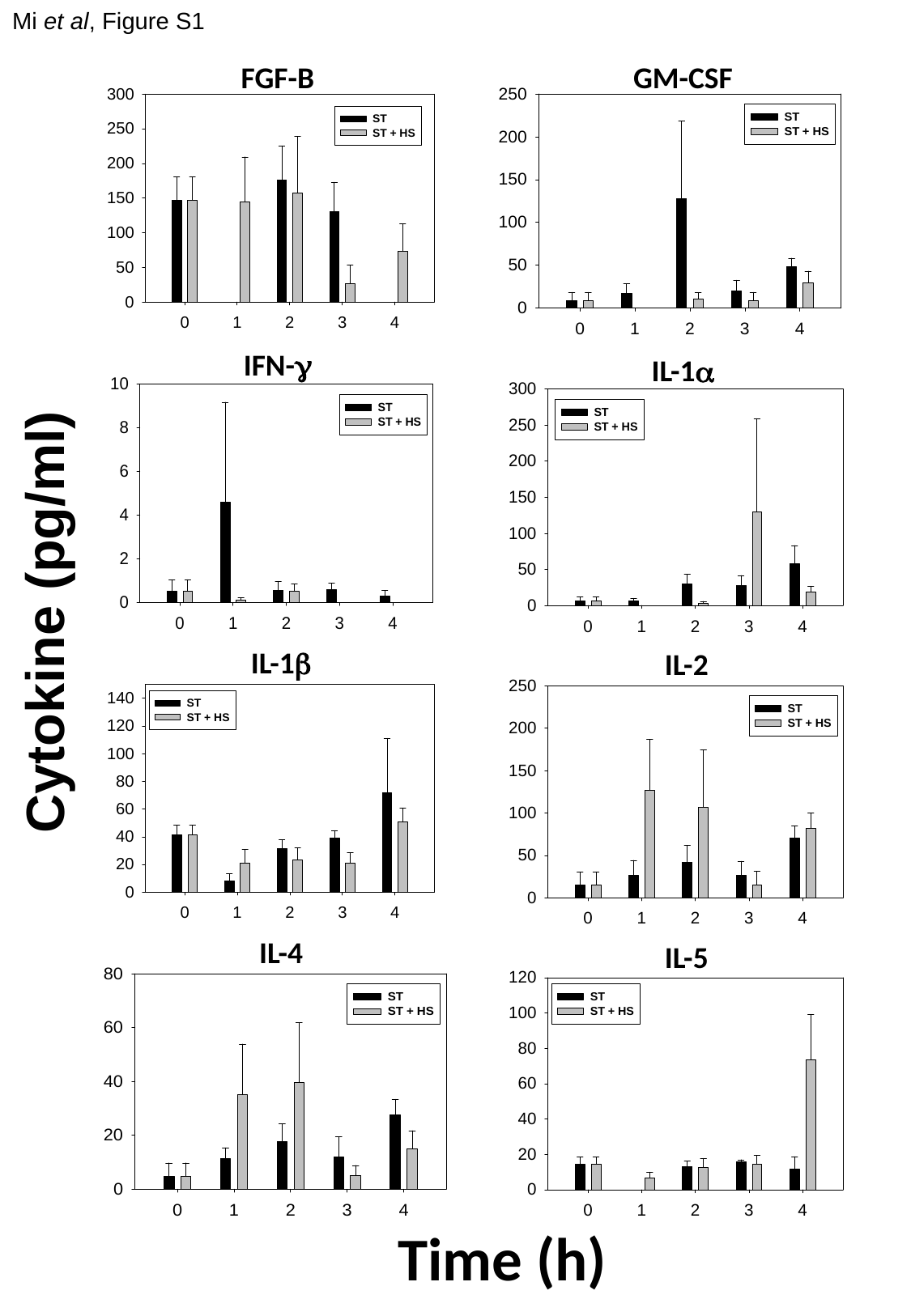

Mi et al, Figure S1
FGF-B
GM-CSF
 IFN-γ
 IL-1α
Cytokine (pg/ml)
 IL-1β
 IL-2
 IL-4
 IL-5
Time (h)

## Slide 2
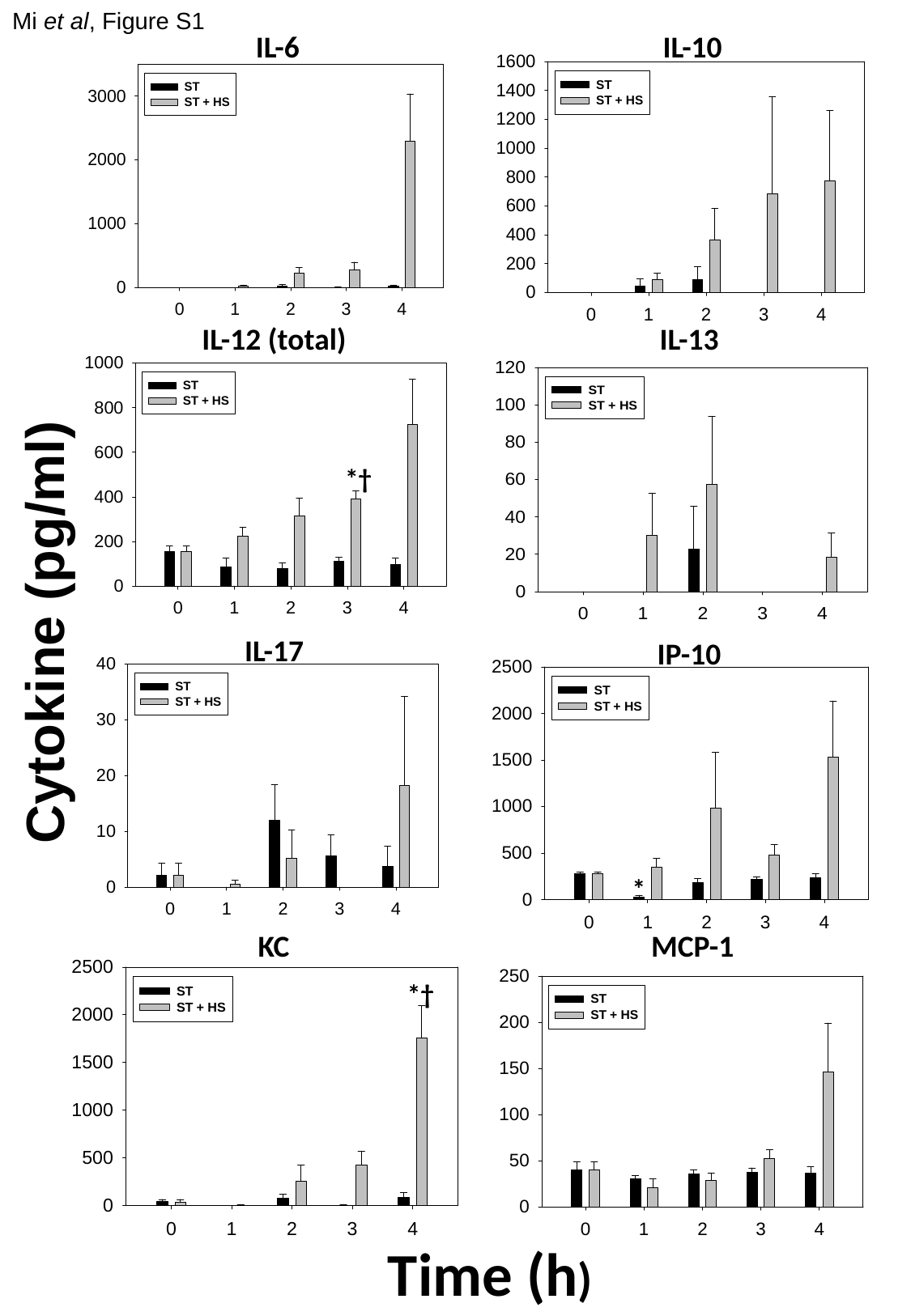

Mi et al, Figure S1
 IL-6
 IL-10
IL-12 (total)
IL-13
*†
Cytokine (pg/ml)
IL-17
IP-10
*
KC
 MCP-1
*†
Time (h)

## Slide 3
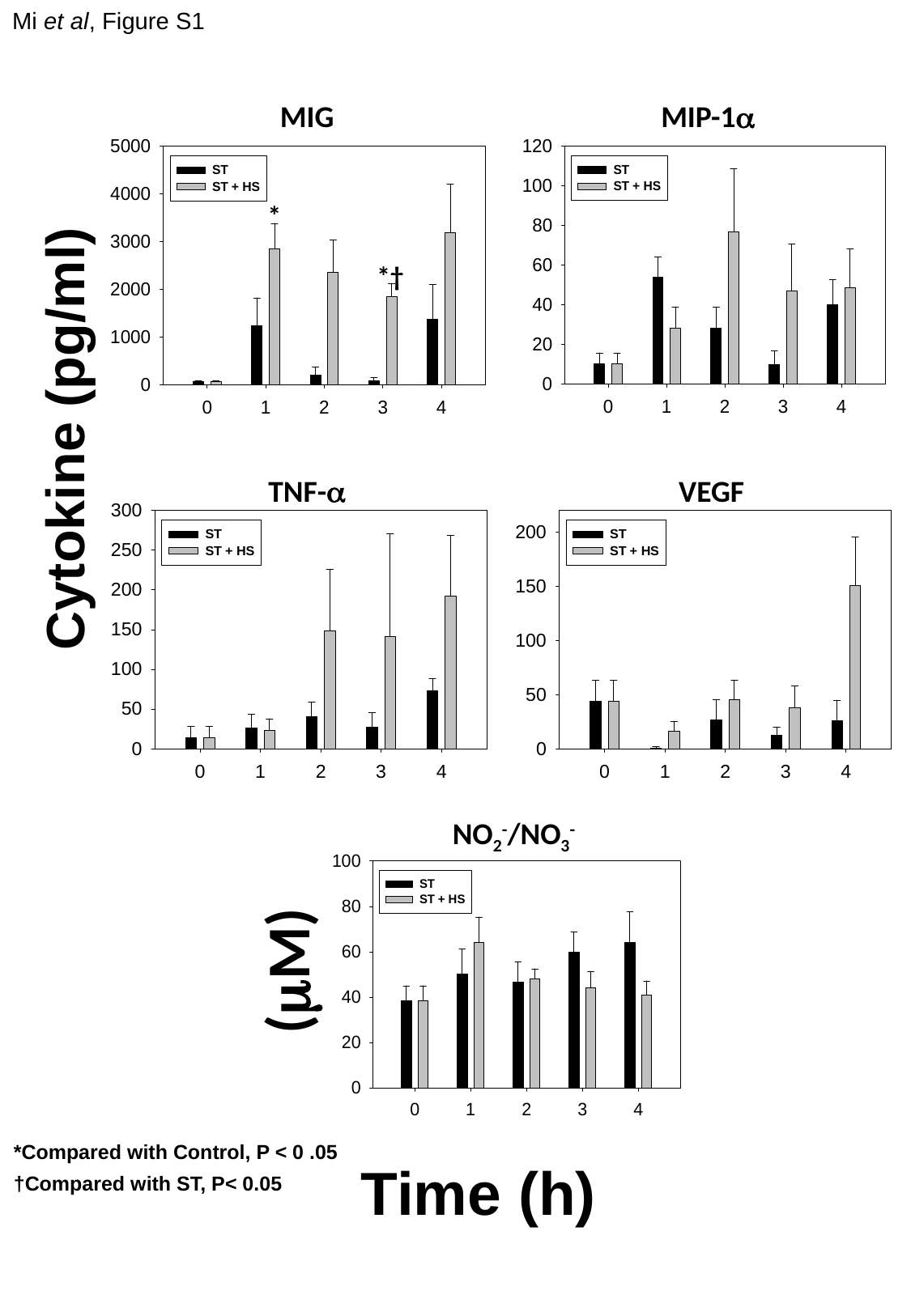

Mi et al, Figure S1
MIG
MIP-1α
*
*†
Cytokine (pg/ml)
 TNF-α
VEGF
NO2-/NO3-
(μM)
*Compared with Control, P < 0 .05
†Compared with ST, P< 0.05
Time (h)
