# Supplementary figures and images for: A Dynamic View of Trauma/Hemorrhage-Induced Inflammation in Mice: Principal Drivers and Networks
Source: PLoS One. 2011 May 10;6(5):e19424. doi: 10.1371/journal.pone.0019424 (PMC3091861; doi:10.1371/journal.pone.0019424)

## Slide 1
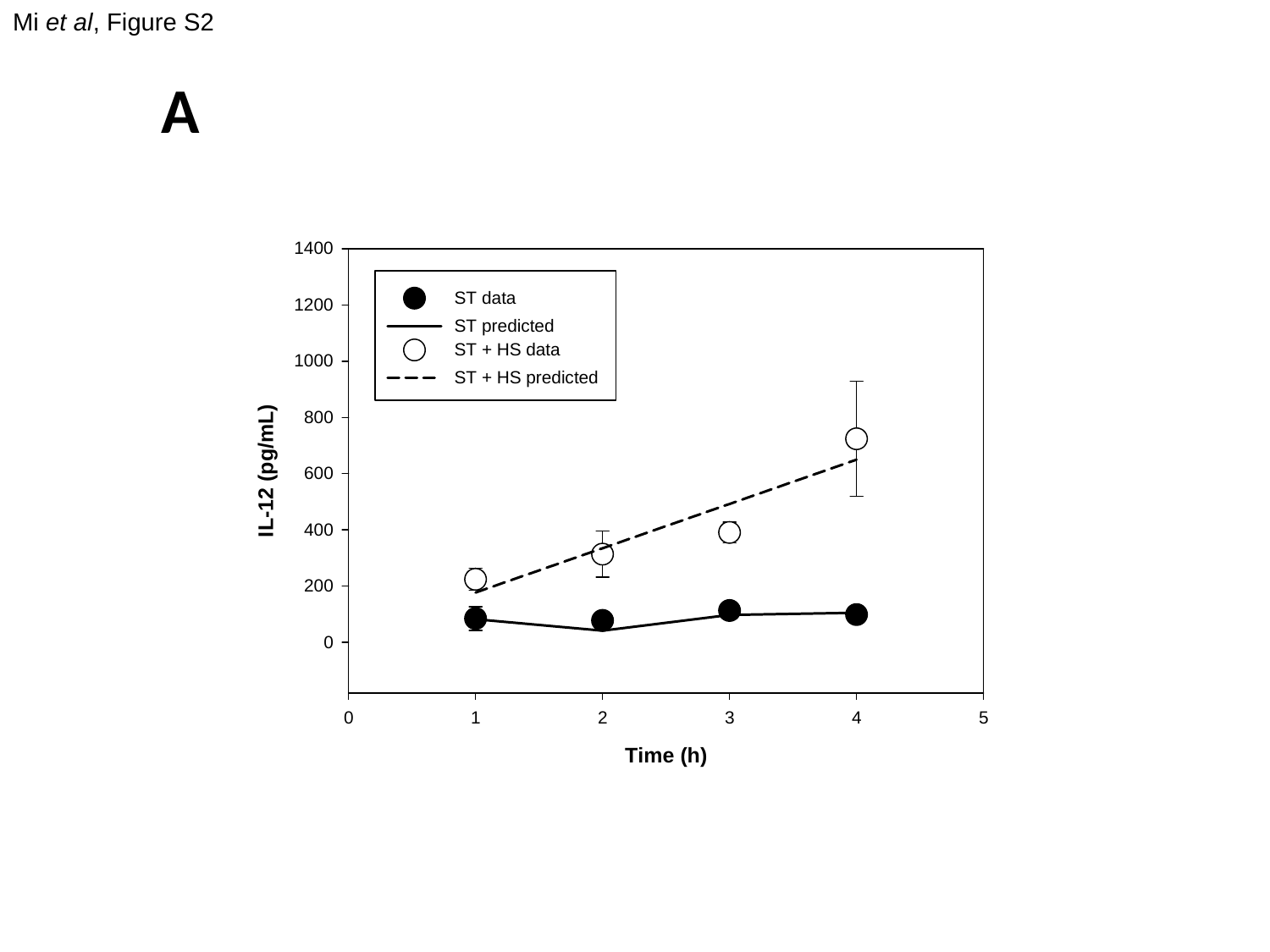

Mi et al, Figure S2
A

## Slide 2
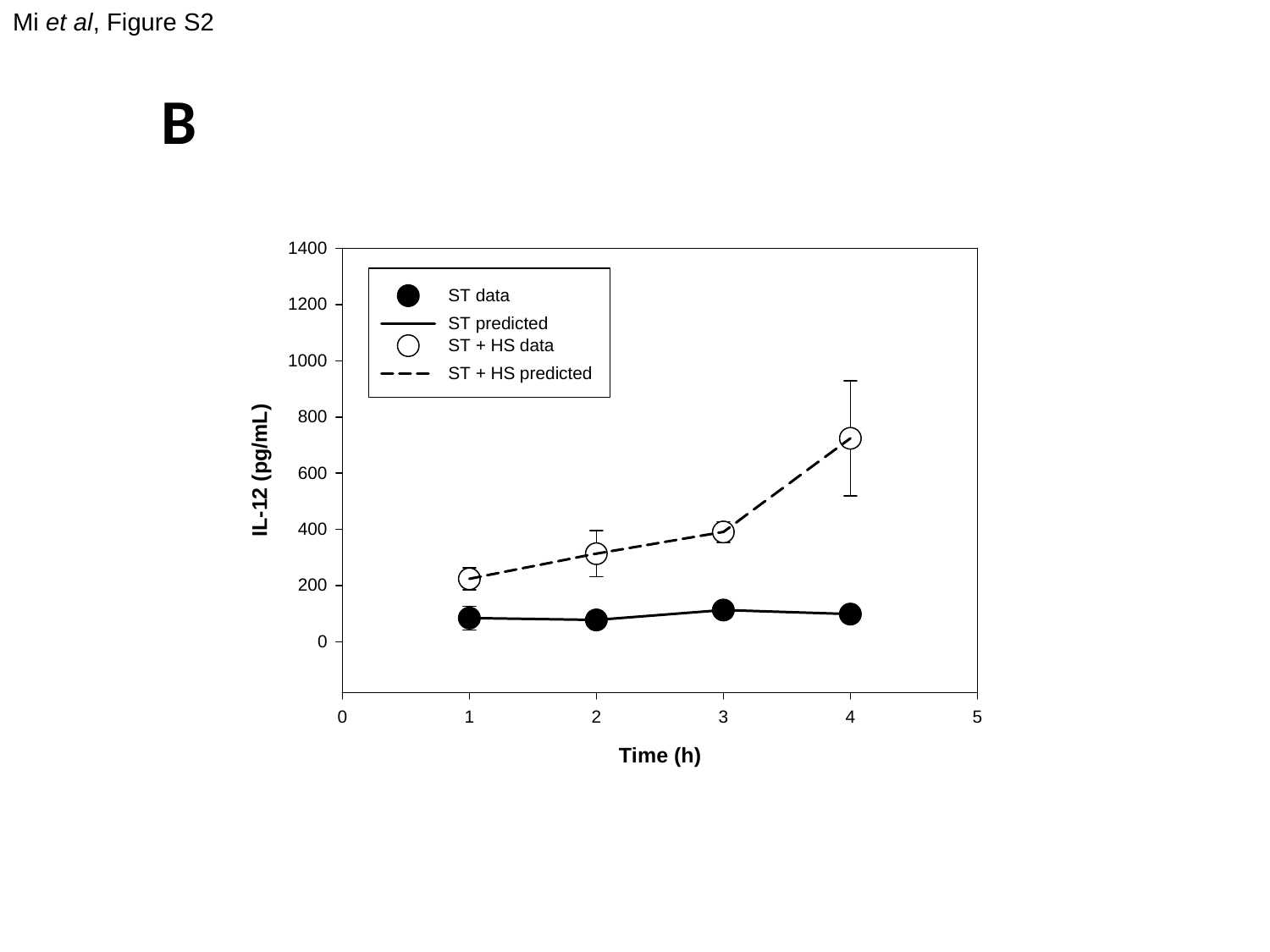

Mi et al, Figure S2
B

## Slide 3
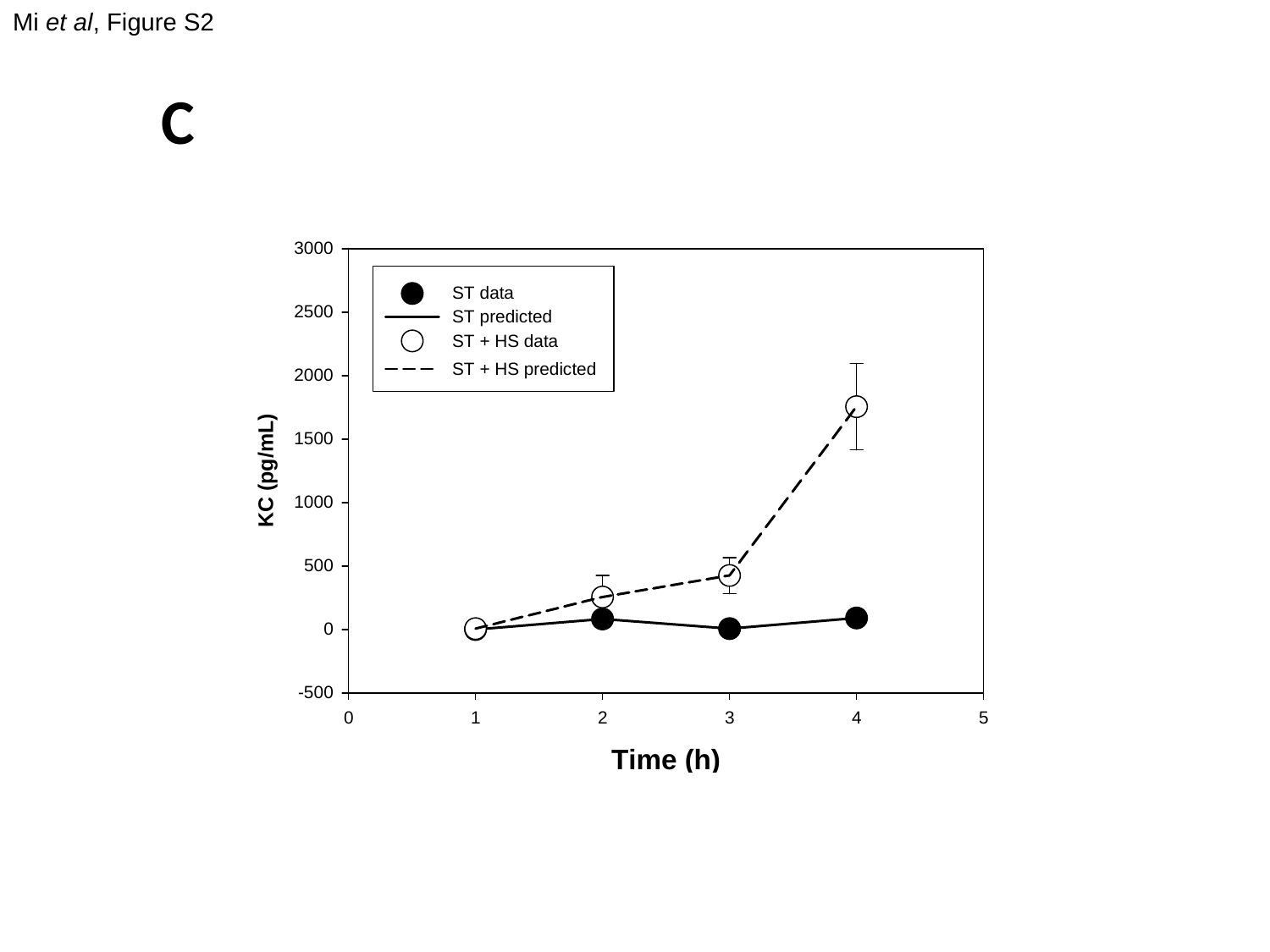

Mi et al, Figure S2
C

## Slide 4
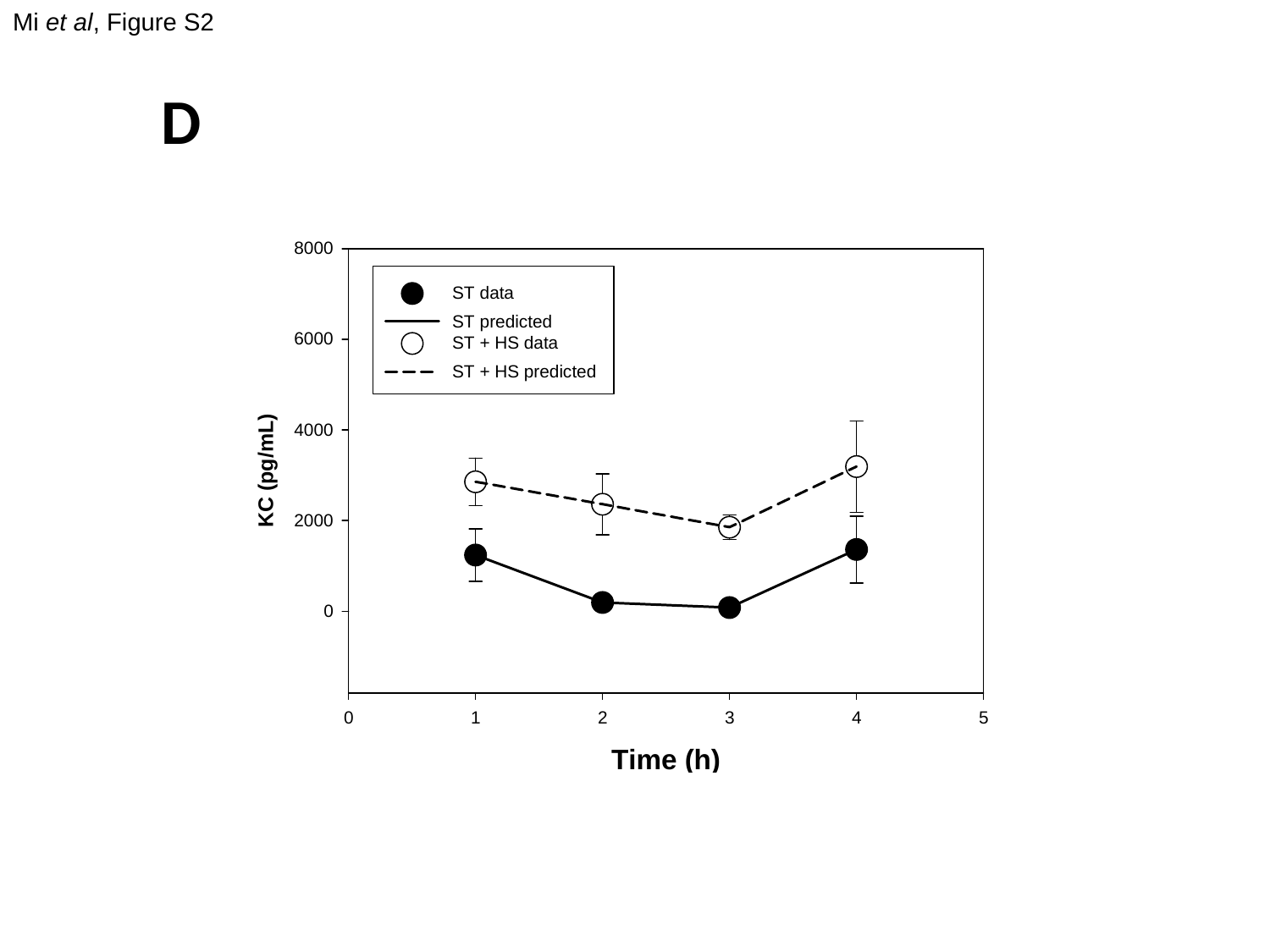

Mi et al, Figure S2
D

Supplement: Figure S2 — Observed and fitted values for IL-12, KC and MIG. Panel A: ANOVA (univariate model) fit for IL-12. Panel B: MNOVA (trivariate model) fit for IL-12. Panel C: MNOVA (trivariate model) fit for KC. Panel D: MNOVA (trivariate model) fit for MIG. (PPT) [file pone.0019424.s002.ppt]
