# Supplementary material for: A Dynamic View of Trauma/Hemorrhage-Induced Inflammation in Mice: Principal Drivers and Networks
Source: PLoS One. 2011 May 10;6(5):e19424. doi: 10.1371/journal.pone.0019424 (PMC3091861; doi:10.1371/journal.pone.0019424)
Supplement: Figure S3 — Additional principal component analyses of ST ± HS. The PCA described in Fig. 3 was repeated, with the number of principal components adjusted to account for 70% (Panels A and C) or 95% (Panels B and D) of the total variance. (PPT) [file pone.0019424.s003.ppt]

## Slide 1
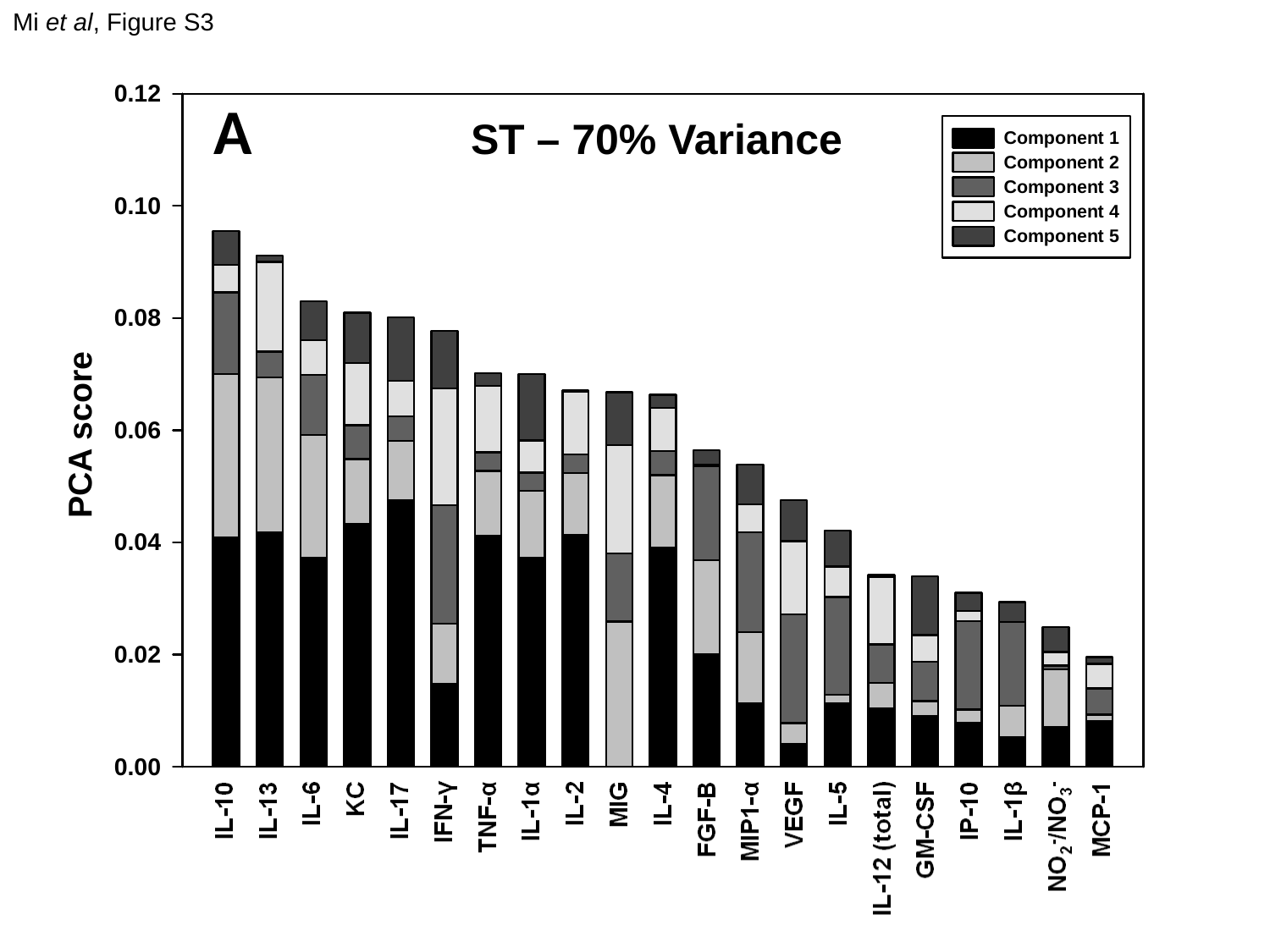

Mi et al, Figure S3
A
ST – 70% Variance

## Slide 2
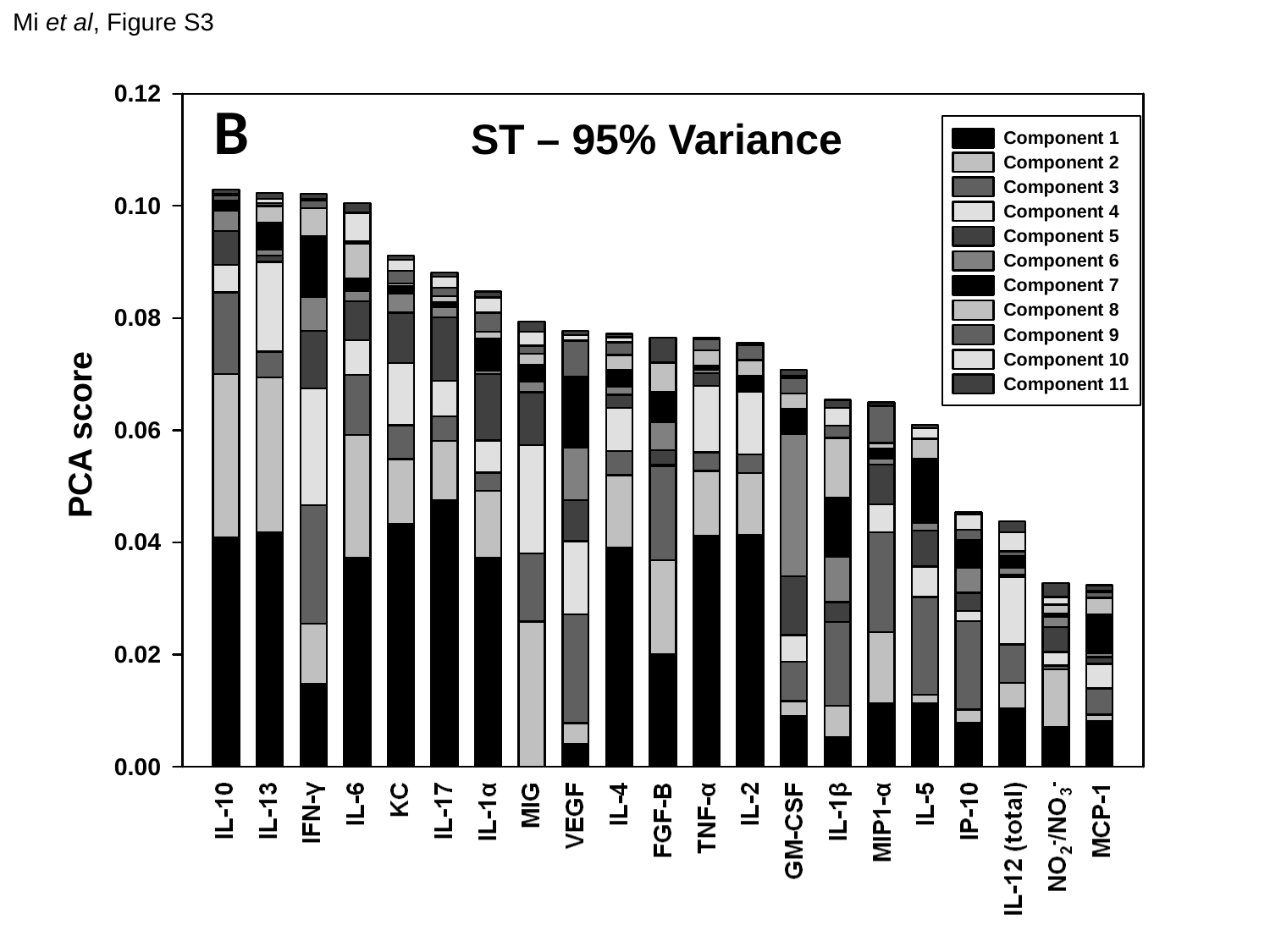

Mi et al, Figure S3
B
ST – 95% Variance

## Slide 3
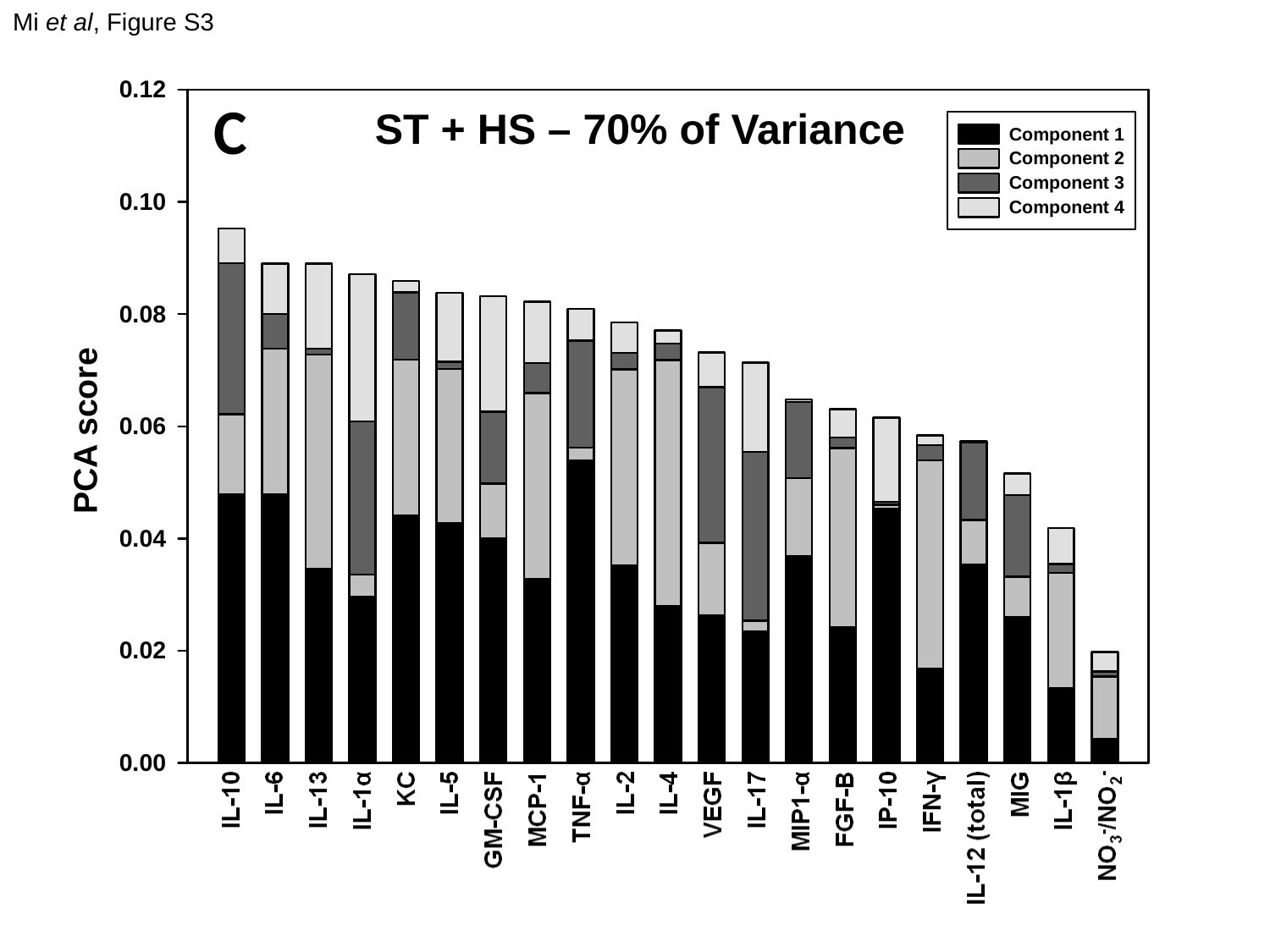

Mi et al, Figure S3
C
ST + HS – 70% of Variance

## Slide 4
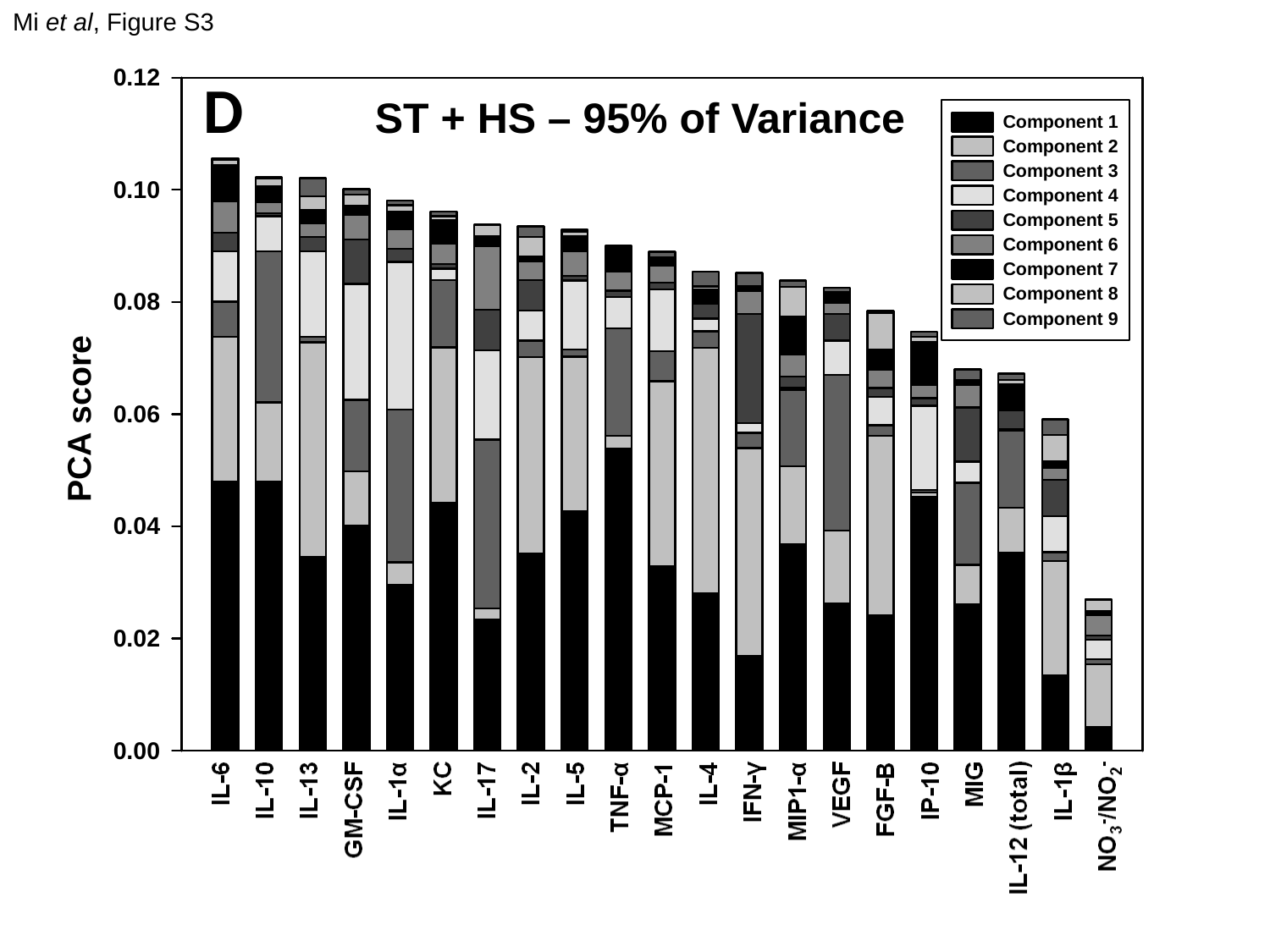

Mi et al, Figure S3
D
ST + HS – 95% of Variance
