# Supplementary material for: A Dynamic View of Trauma/Hemorrhage-Induced Inflammation in Mice: Principal Drivers and Networks
Source: PLoS One. 2011 May 10;6(5):e19424. doi: 10.1371/journal.pone.0019424 (PMC3091861; doi:10.1371/journal.pone.0019424)
Supplement: Figure S4 — Dynamic network analysis of circulating inflammatory mediators following ST ± HS. Mice were subjected to ST ± HS followed by measurement of cytokines, chemokines, and NO2 −/NO3 −, followed by Dynamic Network Analysis as described in the Materials and Methods . Red nodes indicate that the mediator is statistically significantly different from its baseline value (no treatment [time = 0]; p<0.05). White nodes indicate no significant change compared to no treatment (time = 0). Green edges signify a positive correlation and blue edges signify a negative correlation. Panel A: Dynamic networks between 0–1 h. Panel B: Dynamic networks between 1–2 h. Panel C: Dynamic networks between 2–3 h. Panel D: Dynamic networks between 3–4 h. (PPT) [file pone.0019424.s004.ppt]

## Slide 1
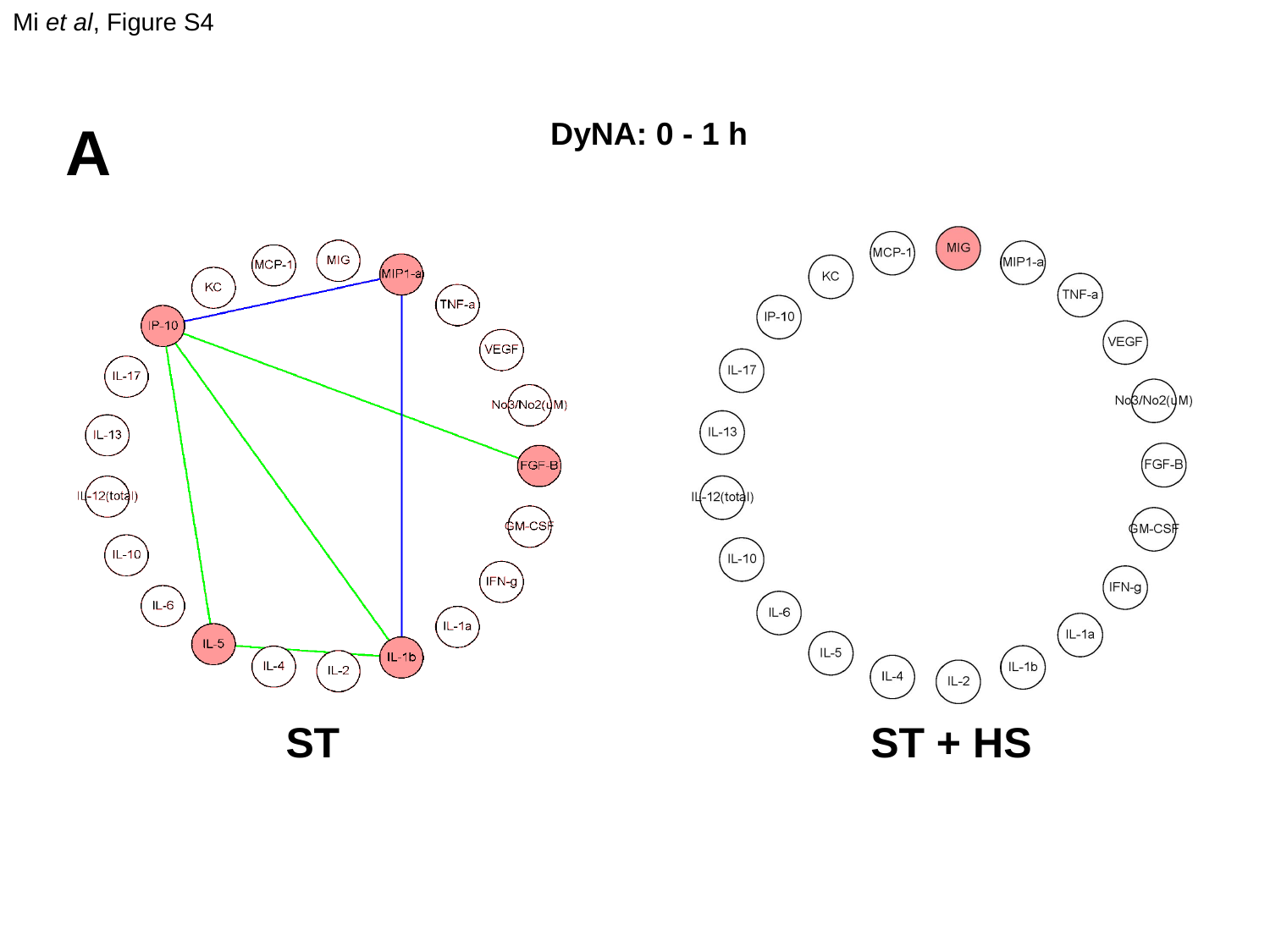

Mi et al, Figure S4
A
 DyNA: 0 - 1 h
ST
ST + HS

## Slide 2
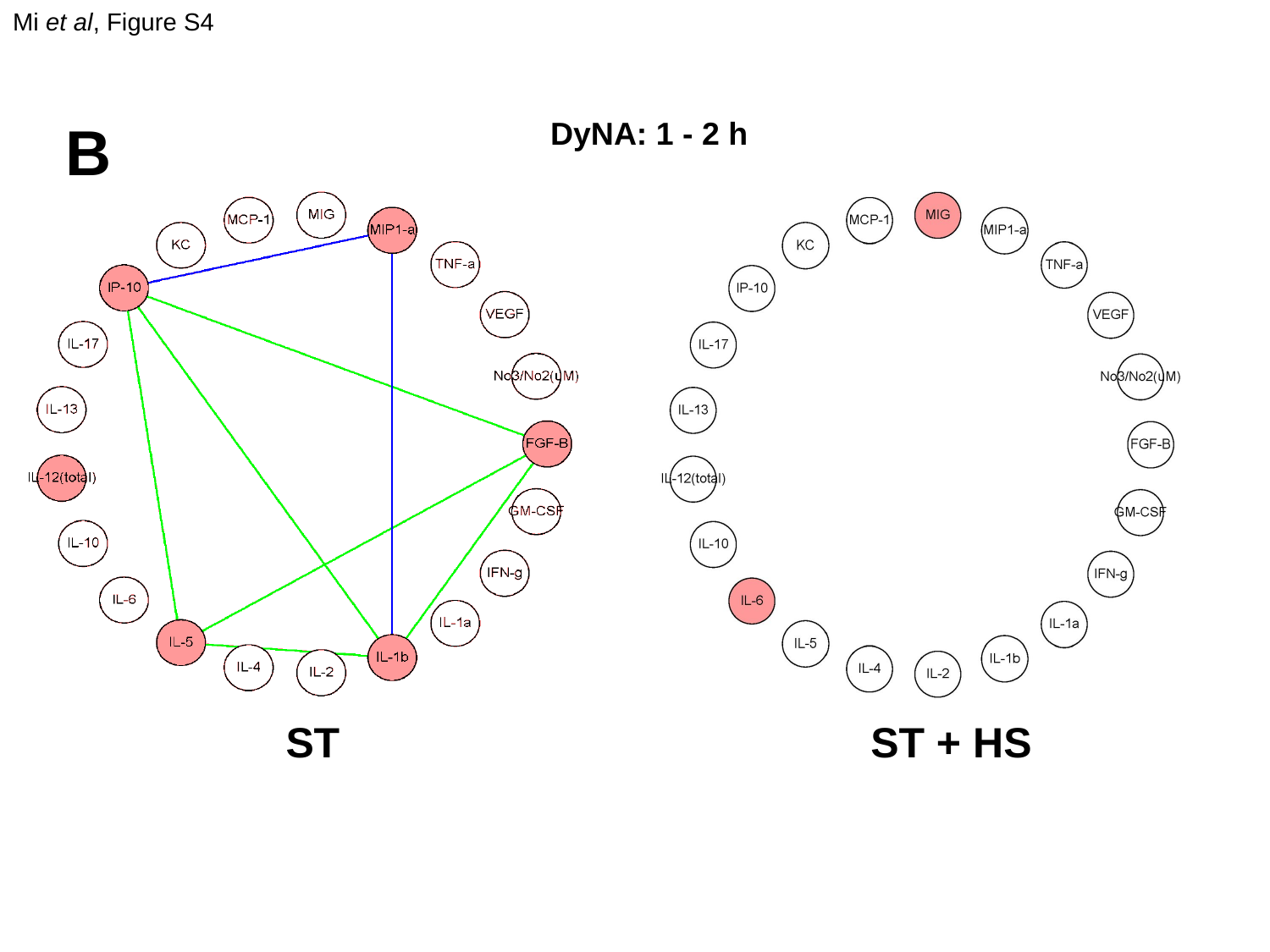

Mi et al, Figure S4
B
 DyNA: 1 - 2 h
ST
ST + HS

## Slide 3
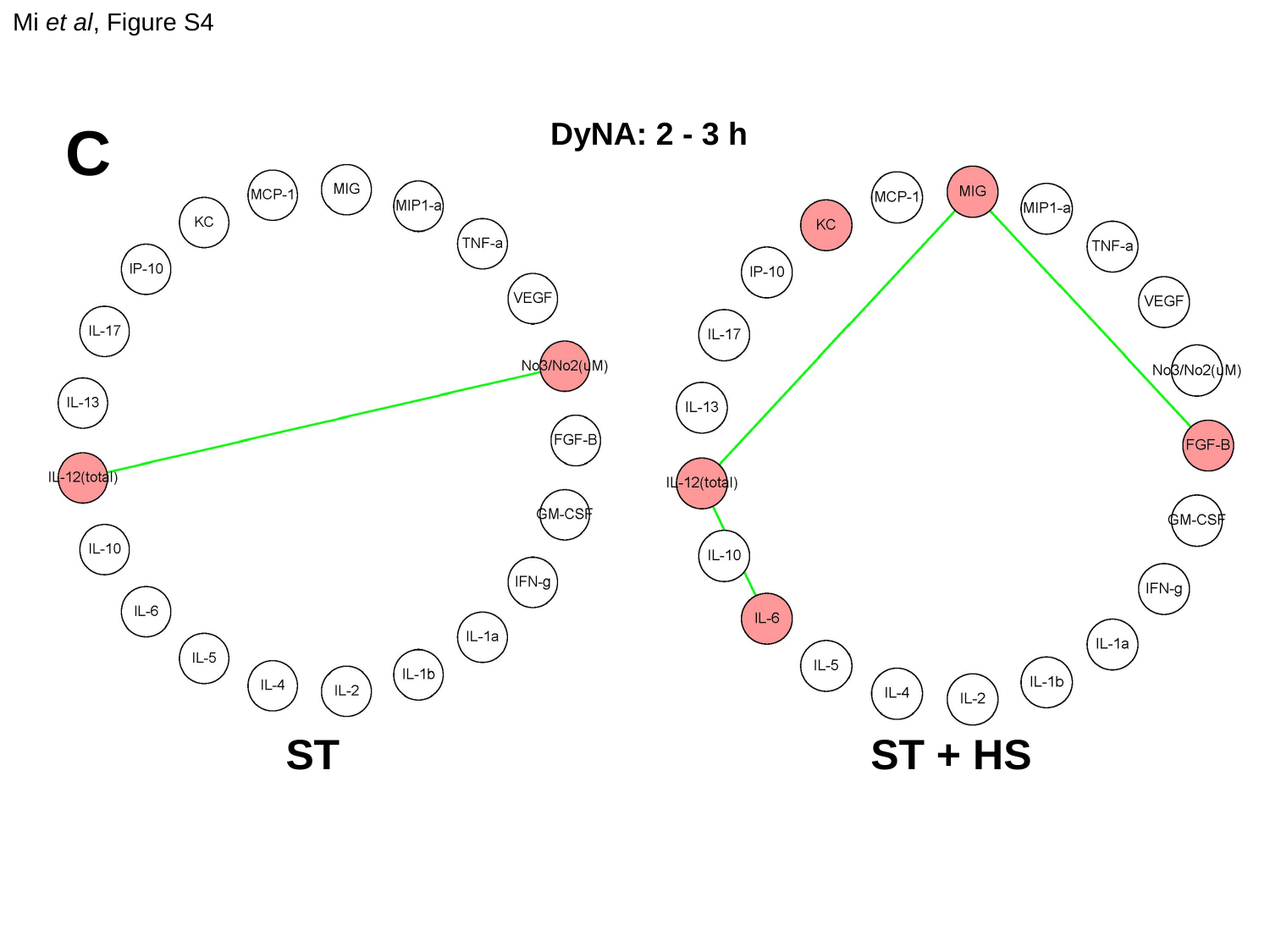

Mi et al, Figure S4
C
 DyNA: 2 - 3 h
ST
ST + HS

## Slide 4
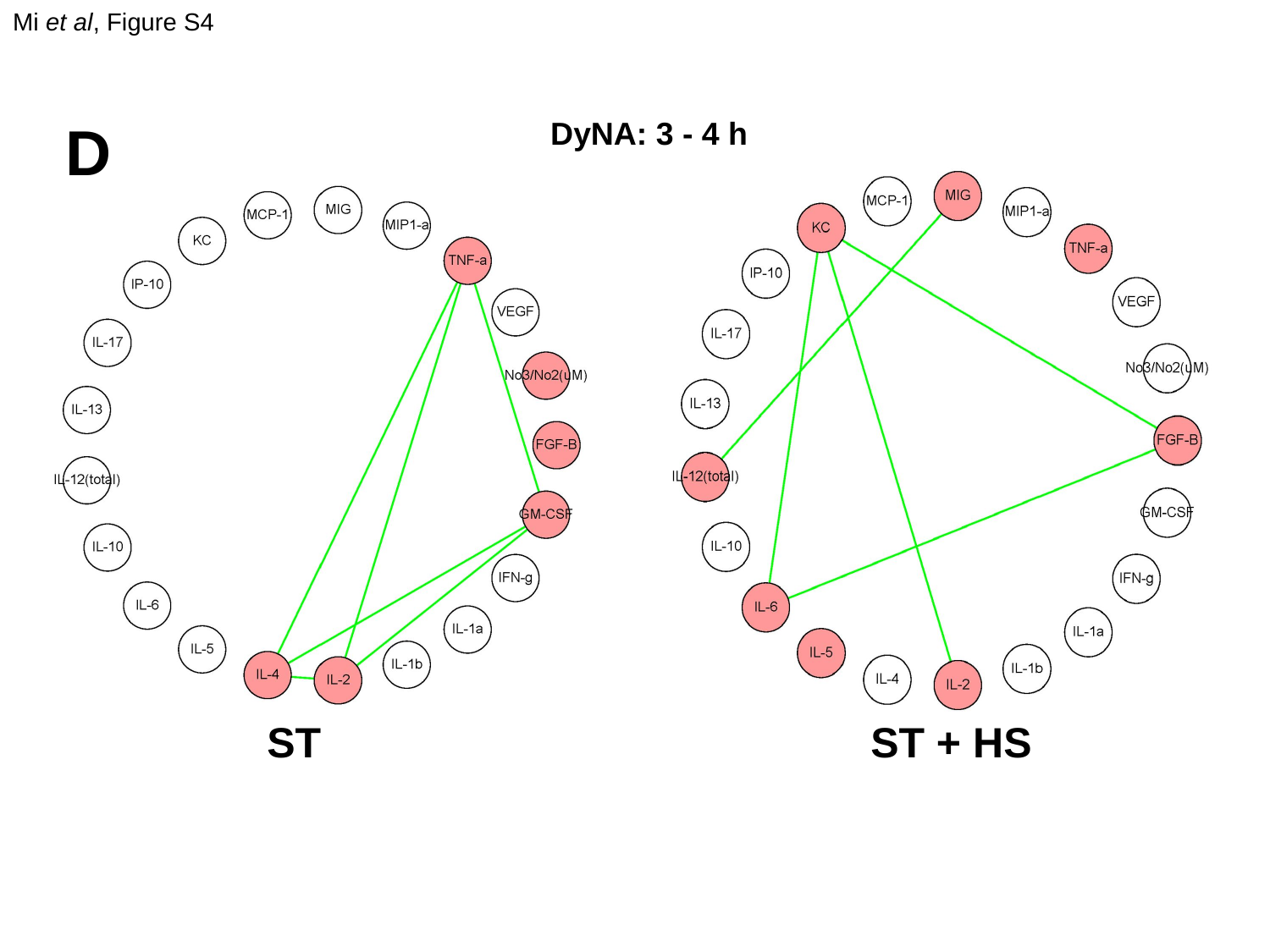

Mi et al, Figure S4
D
 DyNA: 3 - 4 h
ST
ST + HS
