# Supplementary material for: A Dynamic View of Trauma/Hemorrhage-Induced Inflammation in Mice: Principal Drivers and Networks
Source: PLoS One. 2011 May 10;6(5):e19424. doi: 10.1371/journal.pone.0019424 (PMC3091861; doi:10.1371/journal.pone.0019424)
Supplement: Table S1 — Circulating cytokines and chemokines from mice subjected to ST ± HS. Mice were untreated, subjected to ST for the indicated times, or subjected to ST + HS for the indicated times. Serum was obtained following euthanasia and assayed for the indicated cytokines and chemokines using Luminex™ as described in the Materials and Methods . Values are in pg/ml and are given as mean ± SEM. *compared with baseline, P<0.05. †compared with ST, P<0.05. Levene statistic is calculated for variance test and it suggests that the equal variance assumption is rejected with P<0.05. Then one-way ANOVA post Hoc is performed by using Games-Howell test for unequal variances. There are total 9 groups with n = 6 in each group. (DOC) [file pone.0019424.s005.doc]

**Table S1. Circulating cytokines and chemokines from mice subjected to ST ± HS.** Mice were untreated, subjected to ST for the indicated times, or subjected to ST + HS for the indicated times. Serum was obtained following euthanasia and assayed for the indicated cytokines and chemokines using Luminex™ as described in the *Materials and Methods*. Values are in pg/ml and are given as mean ± SEM. *compared with baseline, P < 0.05. †compared with ST, P < 0.05.

Levene statistic is calculated for variance test and it suggests that the equal variance assumption is rejected with P < 0.05. Then one-way ANOVA post Hoc is performed by using Games-Howell test for unequal variances. There are total 9 groups with n=6 in each group

|  | *FGF-B* | *GM-CSF* | *IFN-γ* | *IL-1α* | *IL-1β* | *IL-2* | *IL-4* | *IL-5* | *IL-6* | *IL-10* |
| --- | --- | --- | --- | --- | --- | --- | --- | --- | --- | --- |
| **Controls** | 147.2±80.5 | 9.2±22.7 | 0.5±1.2 | 5.7±14.1 | 41.2±17.3 | 15.2±37.3 | 4.8±11.8 | 14.5±9.6 | 0.0±0.0 | 0.0±0.0 |
| **1 h ST** | 0.0±0.0 | 17.5±27.2 | 4.5±11.2 | 5.8±9.8 | 8.0±12.7 | 27.0±41.8 | 11.3±9.9 | 0.0±0.0 | 0.0±0.0 | 46.7±114.5 |
| **1 h ST + HS** | 144.4±158.7 | 0.0±0.0 | 0.1±0.2 | 0.0±0.0 | 21.2±23.6 | 126.6±147.1 | 35.1±45.8 | 6.6±8.0 | 19.2±26.7 | 91.4±104.5 |
| **2 h ST** | 175.9±120.9 | 128.0±220.6 | 0.5±0.9 | 29.5±33.8 | 31.3±16.0 | 42.4±46.5 | 17.5±16.4 | 13.2±6.7 | 27.9±42.5 | 89.8±220.0 |
| **2 h ST + HS** | 157.0±200.6 | 10.6±18.1 | 0.5±0.8 | 2.9±6.3 | 23.4±20.6 | 106.7±165.5 | 39.5±54.5 | 12.7±12.0 | 225.4±217.4 | 363.7±530.1 |
| **3 h ST** | 130.5±103.2 | 19.9±30.9 | 0.5±0.6 | 28.1±32.9 | 39.3±12.6 | 26.4±40.9 | 11.8±18.3 | 15.9±2.6 | 2.6±6.4 | 0.0±0.0 |
| **3 h ST + HS** | 26.4± 26.4 | 9.2± 22.7 | 0±0 | 129.3± 129.3 | 20.8±7.6 | 15.5±15.5 | 5.1± 3.7 | 14.5±4.9 | 275.3±121.8 | 684± 669.6 |
| **4 h ST** | 0.0±0.0 | 48.1±24.7 | 0.3±0.6 | 58.2±60.2 | 71.7±96.3 | 70.6±34.9 | 27.6±13.9 | 11.9±16.6 | 22.8±32.5 | 0.0±0.0 |
| **4 h ST + HS** | 73.3±96.8 | 29.5±33.2 | 0.0±0.0 | 19.0±18.7 | 50.5±24.9 | 82.0±43.8 | 14.9±16.5 | 73.7±62.3 | 2298.2±1808.0 | 772.2±1199.8 |

**Table S**1 (continued)

|  | *IL-12* | *IL-13* | *IL-17* | *IP-10* | *KC* | *MCP-1* | *MIG* | *MIP1-α* | TNF | VEGF |
| --- | --- | --- | --- | --- | --- | --- | --- | --- | --- | --- |
| **Controls`** | 156.5±55.4 | 0.0±0.0 | 2.1±5.2 | 280.0±41.5 | 39.9±62.0 | 40.4±20.7 | 65.6±32.6 | 10.1±13.4 | 14.0±34.3 | 43.6±47.5 |
| **1 h ST** | 84.8±102.5 | 0.0±0.0 | 0.0±0.0 | 27.5±43.8* | 0.0±0.0 | 30.6±8.3 | 1241.4±1421.4 | 54.0±24.5 | 26.4±41.0 | 0.9±2.3 |
| **1 h ST + HS** | 224.3±96.4 | 30.3±54.8 | 0.5±1.4 | 346.1±231.3 | 6.1±6.8 | 20.8±22.7 | 2857.1±1281.0* | 28.0±26.6 | 23.1±36.5 | 16.4±22.0 |
| **2 h ST** | 77.9±62.3 | 22.8±55.9 | 12.0±15.6 | 182.5±99.2 | 82.3±95.7 | 35.5±11.5 | 196.2±437.0 | 28.2±25.7 | 40.6±44.5 | 26.6±45.7 |
| **2 h ST + HS** | 313.7±199.8 | 57.4±89.0 | 5.1±12.5 | 986.8±1453.9 | 255.1±416.6 | 28.8±19.7 | 2361.1±19.7 | 76.9±77.2 | 148.4±189.8 | 45.5±44.0 |
| **3 h ST** | 113.0±37.9 | 0.0±0.0 | 5.6±9.1 | 219.9±63.1 | 6.3±15.4 | 37.2±12.5 | 83.8±165.7 | 9.9±16.2 | 27.6±43.0 | 12.8±17.3 |
| **3 h ST + HS** | 390.7±37.2*† | 0±0 | 0±0 | 476.6±112.7 | 425.4±141.4 | 52.5±9.2 | 1854.2±270.4*† | 46.9295±23.6 | 141.6±128.9 | 38.1±19.6 |
| **4 h ST** | 98.6±64.9 | 0.0±0.0 | 3.7±8.6 | 232.6±102.8 | 90.6±115.6 | 36.5±16.8 | 1366.3±1804.1 | 40.0±30.8 | 72.8±37.6 | 26.2±46.0 |
| **4 h ST + HS** | 723.8±500.2 | 18.5±31.3 | 18.2±39.0 | 1534.0±1468.0 | 1755.6±834.9*† | 145.8±130.1 | 3192.5±2467.7 | 48.6±47.6 | 191.7±187.7 | 150.5±109.7 |
