# Supplementary material for: A Dynamic View of Trauma/Hemorrhage-Induced Inflammation in Mice: Principal Drivers and Networks
Source: PLoS One. 2011 May 10;6(5):e19424. doi: 10.1371/journal.pone.0019424 (PMC3091861; doi:10.1371/journal.pone.0019424)
Supplement: Table S2 — Circulating NO2−/NO3− values from mice subjected to ST ± HS. Mice were untreated, subjected to ST for the indicated times, or subjected to ST + HS for the indicated times. Serum was obtained following euthanasia and assayed for NO2 −/NO3 − using the nitrate reductase method as described in the Materials and Methods . (DOC) [file pone.0019424.s006.doc]

**Table S2. Circulating NO_2_^-^/NO_3_^-^ values from mice subjected to ST ± HS. Mice were untreated, subjected to ST for the indicated times, or subjected to ST + HS for the indicated times. Serum was obtained following euthanasia and assayed for NO_2_^-^/NO_3_^-^ using the nitrate reductase method as described in the *Materials and Methods*.**

|  | *NO_2_^-^/NO_3_^-^* |
| --- | --- |
| **Controls** | 50.3±26.8 |
| **1 h ST** | 38.3±16.5 |
| **1 h ST + HS** | 64.0±27.5 |
| **2 h ST** | 46.8±19.6 |
| **2 h ST + HS** | 48.2±9.3 |
| **3 h ST** | 59.8±17.8 |
| **3 h ST + HS** | 44±7.3 |
| **4 h ST** | 64.3±32.8 |
| **4 h ST + HS** | 41.0±15.0 |
