# Supplementary material for: A Dynamic View of Trauma/Hemorrhage-Induced Inflammation in Mice: Principal Drivers and Networks
Source: PLoS One. 2011 May 10;6(5):e19424. doi: 10.1371/journal.pone.0019424 (PMC3091861; doi:10.1371/journal.pone.0019424)
Supplement: Table S3 — Univariate analysis of circulating inflammatory mediators following ST and ST + HS. Mice were subjected to ST ± HS followed by measurement of cytokines, chemokines, and NO2 −/NO3 − as described in the Materials and Methods . t-values were calculated for the individual inflammatory mediators at fixed time points (1, 2, 3, and 4 h) and across the entire time range as whole. Since the number of experimental animals for each experimental procedure at each time point is 6, there are 6+6−2 = 10 degrees of freedom for all differences for a fixed time comparison. The last row gives t-values for (mean ST + HS – mean ST) across all time levels; these comparisons carry 24+24 – 2 = 46 degrees of freedom. (DOC) [file pone.0019424.s007.doc]

**Table S3: Univariate analysis of circulating inflammatory mediators following ST and ST + HS.** Mice were subjected to ST ± HS followed by measurement of cytokines, chemokines, and NO_2_^-^/NO_3_^-^ as described in the *Materials and Methods*. t-values were calculated for the individual inflammatory mediators at fixed time points (1, 2, 3, and 4 h) and across the entire time range as whole. Since the number of experimental animals for each experimental procedure at each time point is 6, there are 6+6-2=10 degrees of freedom for all differences for a fixed time comparison. The last row gives t-values for (mean ST + HS – mean ST) across all time levels; these comparisons carry 24 + 24 – 2 = 46 degrees of freedom.

T value Table

|  | *FGF.B* | *GM-CSF* | *IFN-γ* | *IL-1α* | *IL-1β* | *IL-2* | *IL-4* |
| --- | --- | --- | --- | --- | --- | --- | --- |
| **1 h** | 2.23 | -1.58 | -0.98 | -1.46 | 1.20 | 1.60 | 1.24 |
| **2 h** | -0.20 | -1.30 | -0.11 | -1.89 | -0.74 | 0.92 | 0.95 |
| **3 h** | -2.09 | -0.68 | -2.11 | 0.78 | -2.01 | -0.48 | -0.80 |
| **4 h** | 1.85 | -1.09 | -1.22 | -1.53 | -0.52 | 0.50 | -1.44 |
| **Overall** | *0.65* | *-1.72* | *-1.19* | *0.22* | *-0.74* | *1.64* | *0.80* |

|  | *IL-5* | *IL-6* | *IL-10* | *IL-12 total* | *IL-13* | *IL-17* | *IP-10* |
| --- | --- | --- | --- | --- | --- | --- | --- |
| **1 h** | 2.03 | 1.76 | 0.71 | 2.43 | 1.35 | 1.00 | 3.31 |
| **2 h** | -0.09 | 2.18 | 1.17 | 2.76 | 0.81 | -0.84 | 1.35 |
| **3 h** | -0.27 | 2.24 | 1.02 | 6.88 | N/A | -1.50 | 2.22 |
| **4 h** | 2.35 | 3.08 | 1.58 | 3.04 | 1.45 | 0.89 | 2.17 |
| **Overall** | *1.92* | *2.65* | *2.12* | *4.75* | *1.65* | *0.14* | *3.00* |

|  | *KC* | *MCP-1* | *MIG* | *MIP-1α* | *TNF-α* | *VEGF* | *NO_2_^-^/NO_3_^-^* |
| --- | --- | --- | --- | --- | --- | --- | --- |
| **1 h** | 2.20 | -0.98 | 2.07 | -1.76 | -0.15 | 1.71 | 1.78 |
| **2 h** | 0.99 | -0.71 | 3.13 | 1.47 | 1.35 | 0.73 | 0.18 |
| **3 h** | 2.96 | 1.45 | 6.35 | 1.51 | 0.88 | 1.21 | -1.37 |
| **4 h** | 4.84 | 2.04 | 1.46 | 0.37 | 1.52 | 2.56 | -1.58 |
| **Overall** | *3.31* | *1.62* | *4.42* | *1.36* | *1.98* | *2.59* | *-0.51* |

P value Table

|  | *FGF.B* | *GM-CSF* | *IFN-γ* | *IL-1α* | *IL-1β* | *IL-2* | *IL-4* |
| --- | --- | --- | --- | --- | --- | --- | --- |
| **1 h** | P<0.05* | P>0.2 | P>0.2 | P>0.1 | P>0.2 | P>0.1 | P>0.2 |
| **2 h** | P>0.2 | P>0.2 | P>0.2 | P>0.05 | P>0.2 | P>0.2 | P>0.2 |
| **3 h** | P>0.05 | P>0.2 | P>0.05 | P>0.2 | P>0.05 | P>0.2 | P>0.2 |
| **4 h** | P>0.05 | P>0.2 | P>0.2 | P>0.1 | P>0.2 | P>0.2 | P>0.1 |
| **Overall** | *P>0.2* | *P>0.05* | *P>0.2* | *P>0.2* | *P>0.2* | *P>0.1* | *P>0.2* |

|  | *IL-5* | *IL-6* | *IL-10* | *IL-12 total* | *IL-13* | *IL-17* | *IP-10* |
| --- | --- | --- | --- | --- | --- | --- | --- |
| **1 h** | P>0.05 | P>0.1 | P>0.2 | P<0.05* | P>0.2 | P>0.2 | P<0.01 |
| **2 h** | P>0.2 | P>0.05 | P>0.2 | P<0.05* | P>0.2 | P>0.2 | P>0.2 |
| **3 h** | P>0.2 | P<0.05* | P>0.2 | P<0.01* | N/A | P>0.1 | P>0.05 |
| **4 h** | P<0.05* | P<0.05* | P>0.1 | P<0.02* | P>0.2 | P>0.2 | P>0.05 |
| **Overall** | P*>0.05* | *P<0.02** | *P<0.05** | *P<0.01** | *P>0.1* | *P>0.2* | *P<0.01** |

|  | *KC* | *MCP-1* | *MIG* | *MIP-1α* | *TNF-α* | *VEGF* | *NO_2_^-^/NO_3_^-^* |
| --- | --- | --- | --- | --- | --- | --- | --- |
| **1 h** | P>0.05 | P>0.2 | P>0.05 | P>0.1 | P>0.2 | P>0.1 | P>0.1 |
| **2 h** | P>0.2 | P>0.2 | P<0.02* | P>0.1 | P>0.2 | P>0.2 | P>0.2 |
| **3 h** | P<0.02* | P>0.1 | P<0.01* | P>0.1 | P>0.2 | P>0.2 | P>0.2 |
| **4 h** | P<0.01* | P>0.05 | P>0.1 | P>0.2 | P>0.1 | P<0.05* | P>0.1 |
| **Overall** | *P<0.01** | *P>0.1* | *P<.01** | *P>0.1* | *P>0.05* | *P<0.02** | *P>0.2* |
