# Supplementary material for: A Dynamic View of Trauma/Hemorrhage-Induced Inflammation in Mice: Principal Drivers and Networks
Source: PLoS One. 2011 May 10;6(5):e19424. doi: 10.1371/journal.pone.0019424 (PMC3091861; doi:10.1371/journal.pone.0019424)
Supplement: Table S4 — Summary of ANOVA models for five significant mediators. Mice were subjected to ST ± HS followed by measurement of cytokines, chemokines, and NO2 −/NO3 − as described in the Materials and Methods . ANOVA was carried out to determine any interaction effect between Experimental Procedure and Time for all inflammatory mediators studied. (DOC) [file pone.0019424.s008.doc]

**Table S4: Summary of ANOVA models for five significant mediators.** Mice were subjected to ST ± HS followed by measurement of cytokines, chemokines, and NO_2_^-^/NO_3_^-^ as described in the *Materials and Methods*. ANOVA was carried out to determine any interaction effect between Experimental Procedure and Time for all inflammatory mediators studied.

|  | *F-val (Proc)* | *p-val (Proc)* | *F-val (Time)* | *p-val (Time)* | *F-val (Int)* | *p-val (Int)* |
| --- | --- | --- | --- | --- | --- | --- |
| **IL-12.total** | 29.90 | 0.00001 | 3.71 | 0.0192 | 3.28 | 0.0301 |
| **IL-6** | 13.45 | 0.0007 | 8.14 | 0.0002 | 7.93 | 0.0002 |
| **IP-10** | 9.74 | 0.0033 | 1.99 | 0.1310 | 1.28 | 0.2925 |
| **KC** | 30.31 | 0.00001 | 15.71 | 0.00001 | 13.40 | 0.00001 |
| **MIG** | 19.98 | 0.0001 | 2.27 | 0.0955 | 0.08 | 0.9712 |
