# Supplementary material for: A Dynamic View of Trauma/Hemorrhage-Induced Inflammation in Mice: Principal Drivers and Networks
Source: PLoS One. 2011 May 10;6(5):e19424. doi: 10.1371/journal.pone.0019424 (PMC3091861; doi:10.1371/journal.pone.0019424)
Supplement: Table S5 — Correlation matrix of the 5 most important mediators. (DOC) [file pone.0019424.s009.doc]

**TABLE S5: Correlation matrix of the 5 most important mediators**

|  | *IL-6* | *IL-12..total*. | *IP-10* | *KC* | *MIG* |
| --- | --- | --- | --- | --- | --- |
| **IL-6** | 1.00 | 0.64 | 0.59 | 0.91 | 0.34 |
| **IL-12..total.** | 0.64 | 1.00 | 0.71 | 0.73 | 0.62 |
| **IP-10** | 0.59 | 0.71 | 1.00 | 0.50 | 0.44 |
| **KC** | 0.91 | 0.73 | 0.50 | 1.00 | 0.46 |
| **MIG** | 0.34 | 0.62 | 0.44 | 0.46 | 1.00 |
