# Supplementary material for: A Dynamic View of Trauma/Hemorrhage-Induced Inflammation in Mice: Principal Drivers and Networks
Source: PLoS One. 2011 May 10;6(5):e19424. doi: 10.1371/journal.pone.0019424 (PMC3091861; doi:10.1371/journal.pone.0019424)
Supplement: Table S6 — Partition of inflammatory mediators by p-values. (DOC) [file pone.0019424.s010.doc]

**Table S6: Partition of inflammatory mediators by p-values.**

| **Cytokine Name** | *IL-12.total* | *MIG* | *IP-10* | BLAH  BBBB | *KC* | *VEGF* | BLAH  BBBB | *IL-6* |
| --- | --- | --- | --- | --- | --- | --- | --- | --- |
| **t-Value** | -3.3 | -3.2 | -3.0 | BBBB | -2.2 | -2.2 | BBBB | -1.9 |
| **p-Value** | 0.00048 | 0.00069 | 0.00135 | BBBB | 0.01390 | 0.01390 | BBBB | 0.02872 |

| **Cytokine Name** | *IL-5* | *TNF-α* | *IL-2* | *IL-13* | *IL-10* | *MIP-1α* | *IFN-γ* | *GM.CSF* |
| --- | --- | --- | --- | --- | --- | --- | --- | --- |
| **t-Value** | -1.7 | -1.6 | -1.54 | -1.46 | -1.42 | -1.31 | 1.32 | 2.2 |
| **p-Value** | 0.04457 | 0.05480 | 0.06178 | 0.07215 | 0.07780 | 0.09510 | 0.90658 | 0.98610 |
