# Supplementary material for: A Dynamic View of Trauma/Hemorrhage-Induced Inflammation in Mice: Principal Drivers and Networks
Source: PLoS One. 2011 May 10;6(5):e19424. doi: 10.1371/journal.pone.0019424 (PMC3091861; doi:10.1371/journal.pone.0019424)
Supplement: Table S7 — The probabilities of correct identification of the ST procedure, based on the logistic model. Mice were subjected to ST ± HS followed by measurement of cytokines, chemokines, and NO2 −/NO3 − as described in the Materials and Methods . Logistic model was created based on the levels of these inflammatory mediators. The table depicts the prediction by this model of the Experimental Procedure. Starred entries show misclassification. (DOC) [file pone.0019424.s011.doc]

**Table S7: The probabilities of correct identification of the ST procedure, based on the logistic model.** Mice were subjected to ST ± HS followed by measurement of cytokines, chemokines, and NO_2_^-^/NO_3_^-^ as described in the *Materials and Methods*. Logistic model was created based on the levels of these inflammatory mediators. The table depicts the prediction by this model of the Experimental Procedure. Starred entries show misclassification.

| **Procedure** | *ST+HS* | *ST+HS* | *ST+HS* | *ST+HS* | *ST+HS* | *ST+HS* | *ST+HS* | *ST+HS* | *ST+HS* |
| --- | --- | --- | --- | --- | --- | --- | --- | --- | --- |
| **Prob** | 0.02 | 0.01 | 0.00 | 0.00 | 0.00 | 0.00 | 0.92* | 0.49* | 0.00 |

| **Procedure** | *ST+HS* | *ST+HS* | *ST+HS* | *ST+HS* | *ST+HS* | *ST+HS* | *ST+HS* | *ST+HS* | *ST+HS* |
| --- | --- | --- | --- | --- | --- | --- | --- | --- | --- |
| **Prob** | 0.00 | 0.00 | 0.00 | 0.37 | 0.00 | 0.00 | 0.00 | 0.00 | 0.00 |

| **Procedure** | *ST+HS* | *ST+HS* | *ST+HS* | *ST+HS* | *ST+HS* | *ST+HS* |
| --- | --- | --- | --- | --- | --- | --- |
| **Prob** | 0.00 | 0.33 | 0.00 | 0.00 | 0.00 | 0.00 |

| **Procedure** | *ST* | *ST* | *ST* | *ST* | *ST* | *ST* | *ST* | *ST* | *ST* |
| --- | --- | --- | --- | --- | --- | --- | --- | --- | --- |
| **Prob** | 0.91 | 1.00 | 0.99 | 0.95 | 1.00 | 0.99 | 0.93 | 0.99 | 0.88 |

| **Procedure** | *ST* | *ST* | *ST* | *ST* | *ST* | *ST* | *ST* | *ST* | *ST* |
| --- | --- | --- | --- | --- | --- | --- | --- | --- | --- |
| **Prob** | 0.98 | 0.97 | 0.92 | 0.98 | 0.99 | 0.96 | 0.97 | 0.92 | 0.95 |

| **Procedure** | *ST* | *ST* | *ST* | *ST* | *ST* | *ST* |
| --- | --- | --- | --- | --- | --- | --- |
| **Prob** | 0.78 | 0.94 | 0.93 | 0.16* | 0.91 | 0.84 |
