# Supplementary material for: A Dynamic View of Trauma/Hemorrhage-Induced Inflammation in Mice: Principal Drivers and Networks
Source: PLoS One. 2011 May 10;6(5):e19424. doi: 10.1371/journal.pone.0019424 (PMC3091861; doi:10.1371/journal.pone.0019424)
Supplement: Document S1 — Detailed experimental description. (DOC) [file pone.0019424.s012.doc]

**SUPPLEMENTARY MATERIALS**

**Detailed Experimental Description**

The cannulation procedure was carried out as follows. Under sterile conditions, both femoral arteries were dissected and cannulated with PA-10 polyethylene tubing (Becton Dickinson 7 Circle, Sparks, MD). Catheters were connected as follows. One was connected to a high frequency blood pressure analyzer (BPA-400, MicroMed, Louisville, KY) for animal monitoring and data gathering. In mice subjected to HS, the other catheter was connected to a motorized, two-way peristaltic pump (Model 720, Instech Labs, Plymouth Meeting, PA) for bleeding and shed blood re-infusion. Shed blood was collected in a sterile syringe container that was placed over a scientific scale (PS602-S, Mettler-Toledo Columbus, OH); the re-infusion of blood was performed from this same reservoir. A separate, sterile reservoir was used for each experimental animal. Animals were placed on a controlled heating pad at 37 C° (Homeothermic Blanket System –Small, Harvard Apparatus Holliston, MA) throughout the experiment. Catheters were secured with tape to the surgical platform and flushed with a total of 2 IU of heparin in saline solution to keep the lines without blood clotting.

Baseline HR, PP, systolic and diastolic pressures, and MAP values were collected over a 15-min period. Bleeding was performed, using a modification of the Wiggers method, to a targeted MAP of 25 mmHg over a 15-min period. Blood pressure was kept at this level for 1, 2, 3 and 4 h, respectively, for most animals. Animals were euthanized at the end of the hypotensive period, and serum was obtained by cardiac puncture for analysis of cytokines (see below). The bleed volume was calculated from changes in blood weight during the experiment. All the animals were kept under sedation using pentobarbital as needed. Sham groups were subjected only to surgical preparation and observed under anesthesia for 1, 2, 3 or 4 h, respectively.

Mean arterial pressure (MAP) was sustained by a computer-controlled re-infusion and withdrawal of shed blood during the experiment by the feedback loop system. During all of these experiments, the various devices were interfaced with a desktop computer (Dell Dimension 4700 Pentium 4 processor 530 with HT Technology [3.00 GHz, 800 FSB] Desktop Computer) for continuous hemodynamic monitoring, data recording and processing. A computerized feedback control loop system was created (depicted graphically in ). The instruments were connected as follows. The scientific scale was connected to a RS-232 Serial Port Card (PCI) DB25 (Quatech, Hudson, OH) and integrated to the computer thought a serial port card DAQCard PCI-6052E for Windows (National Instruments, Austin, TX). The blood pressure analyzer was connected to a SCB-68 Pin shielded I/O connector block (National Instruments, Austin, TX) in a CA-1000 signal conditioning enclosure (National Instruments, Austin, TX). The automated peristaltic pump was integrated to the feedback loop system through a SCB-68 Pin shielded I/O connector block (National Instruments, Austin, TX) and running in an external control mode. A software package was custom-written to set up and control bleeding and resuscitation parameters using this equipment, as described below.

The software program to control HS was written in the LabVIEW™ environment (National Instruments, Austin, TX). Once the program is running (after calibration and testing of all devices), the automated pump performs the initial bleeding to reach the targeted MAP (in the present studies, 25 mmHg). The system calculates the rate of bleeding in order to bleed the mouse to a defined MAP in a given period of time (the RAMP time), which in this study was set to 15 min. Once the animal has been bled to the desired MAP, the system starts to run the second experimental event that keeps the animal at the desired MAP (in these studies, 25 mmHg). All data values are exported in text format files and may be plotted individually. Blood pressure is kept by constant withdraw and re-infusion of the shed blood, maintaining MAP in a range of ±2 mm of Hg. The automated system allows for setting the experimental hypotensive period. After the experiment time is reached, the resuscitation procedure may start as another event.
